# Neuropilin-1/GIPC1 Signaling Regulates α5β1 Integrin Traffic and Function in Endothelial Cells

**DOI:** 10.1371/journal.pbio.1000025

**Published:** 2009-01-27

**Authors:** Donatella Valdembri, Patrick T Caswell, Kurt I Anderson, Juliane P Schwarz, Ireen König, Elena Astanina, Francesca Caccavari, Jim C Norman, Martin J Humphries, Federico Bussolino, Guido Serini

**Affiliations:** 1 Department of Oncological Sciences and Division of Molecular Angiogenesis, Institute for Cancer Research and Treatment, University of Torino School of Medicine, Candiolo, Italy; 2 Beatson Institute for Cancer Research, Bearsden, Glasgow, United Kingdom; 4 Wellcome Trust Centre for Cell-Matrix Research, Faculty of Life Sciences, University of Manchester, Manchester, United Kingdom; 3 Center for Complex Systems in Molecular Biology and Medicine, University of Torino, Torino, Italy; Wellcome Trust Sanger Institute, United Kingdom

## Abstract

Neuropilin 1 (Nrp1) is a coreceptor for vascular endothelial growth factor A165 (VEGF-A165, VEGF-A164 in mice) and semaphorin 3A (SEMA3A). Nevertheless, *Nrp1* null embryos display vascular defects that differ from those of mice lacking either VEGF-A164 or Sema3A proteins. Furthermore, it has been recently reported that Nrp1 is required for endothelial cell (EC) response to both VEGF-A165 and VEGF-A121 isoforms, the latter being incapable of binding Nrp1 on the EC surface. Taken together, these data suggest that the vascular phenotype caused by the loss of *Nrp1* could be due to a VEGF-A164/SEMA3A-independent function of Nrp1 in ECs, such as adhesion to the extracellular matrix. By using RNA interference and rescue with wild-type and mutant constructs, we show here that Nrp1 through its cytoplasmic SEA motif and independently of VEGF-A165 and SEMA3A specifically promotes α5β1-integrin-mediated EC adhesion to fibronectin that is crucial for vascular development. We provide evidence that Nrp1, while not directly mediating cell spreading on fibronectin, interacts with α5β1 at adhesion sites. Binding of the homomultimeric endocytic adaptor GAIP interacting protein C terminus, member 1 (GIPC1), to the SEA motif of Nrp1 selectively stimulates the internalization of active α5β1 in Rab5-positive early endosomes. Accordingly, GIPC1, which also interacts with α5β1, and the associated motor myosin VI (Myo6) support active α5β1 endocytosis and EC adhesion to fibronectin. In conclusion, we propose that Nrp1, in addition to and independently of its role as coreceptor for VEGF-A165 and SEMA3A, stimulates through its cytoplasmic domain the spreading of ECs on fibronectin by increasing the Rab5/GIPC1/Myo6-dependent internalization of active α5β1. Nrp1 modulation of α5β1 integrin function can play a causal role in the generation of angiogenesis defects observed in *Nrp1* null mice.

## Author Summary

The vascular system is a hierarchical network of blood vessels lined by endothelial cells that, by means of the transmembrane integrin proteins, bind to the surrounding proteinaceous extracellular matrix (ECM). Integrins are required for proper cardiovascular development and exist in bent (inactive) and extended (active) shapes that are correspondingly unable and able to attach to the ECM. Extracellular guidance cues, such as vascular endothelial growth factor and semaphorins, bind the transmembrane protein neuropilin-1 (Nrp1) and then activate biochemical signals that, respectively, activate or inactivate endothelial integrins. Here, we show that Nrp1, via its short cytoplasmic domain and independently of vascular endothelial growth factor and semaphorins, specifically promotes endothelial cell attachment to the ECM protein fibronectin, which is known to be crucial for vascular development. Notably, Nrp1 favors cell adhesion by associating with fibronectin-binding integrins and promoting the fast vesicular traffic of their extended form back and forth from the endothelial cell-to-ECM contacts. Binding of the Nrp1 cytoplasmic domain with the adaptor protein GIPC1, which in turn associates with proteins required for integrin internalization and vesicle motility, is required as well. It is likely that such an integrin treadmill could act as a major regulator of cell adhesion in general.

## Abstract 3

The transmembrane protein neuropilin-1 promotes endothelial cell attachment to the extracellular matrix by enhancing active integrin treadmilling at cell-adhesion sites.

## Introduction

In vertebrates, the development of a hierarchically organized and functional vascular tree relies on the dynamic interaction of endothelial cells (ECs) with the surrounding extracellular matrix (ECM), which is mediated by heterodimeric αβ integrin adhesive receptors [1]. During evolution, vertebrates have acquired an additional set of adhesion-related genes that regulate blood vessel assembly and function [2]. Among these genes, the ECM protein fibronectin (FN) and α5β1 integrin, the predominant FN receptor, have proven to be essential for embryonic vascular development and tumor angiogenesis [3]. Indeed, in vertebrate embryos FN is the earliest and most abundantly expressed subendothelial matrix molecule [3,4]. Endothelial α5β1 mediates cell adhesion to FN and the assembly of soluble FN dimers (sFN) into a fibrillar network [3], which has also been implicated in branching morphogenesis [5].

The biological activities of integrins depend on the dynamic regulation of their adhesive function in space and time. In cells, integrins exist in different conformations that determine their affinities for ECM proteins [6] and are continuously endocytosed, trafficked through endosomal compartments, and recycled back to the plasma membrane [7,8]. Therefore, during vascular morphogenesis, real-time modulation of EC–ECM adhesion can result from two interconnected phenomena: the regulation of integrin conformation and traffic in response to extracellular stimuli [8,9]. Indeed, there is mounting evidence that pro- and antiangiogenic cues regulate blood vessel formation by modulating integrin function [1]. In this respect, the transmembrane glycoprotein neuropilin 1 (Nrp1), which is expressed in both neurons and ECs [10], is remarkable because it was originally identified as a surface protein mediating cell adhesion [11] and then found to also act as a coreceptor for both pro- and antiangiogenic factors, such as vascular endothelial growth factor A 165 (VEGF-A165, VEGF-A164 in mice) [12,13] and semaphorin 3A (SEMA3A) [14–20], respectively.

The extracellular region of Nrp1 contains two repeated complement-binding domains (CUB domains; a1-a2 domains), two coagulation-factor-like domains (b1-b2 domains), and a juxtamembrane meprin/A5/μ-phosphatase (MAM; c) homology domain. The Nrp1 intracellular region is only 50 amino acids in length, and its function is poorly characterized [21]. Through its b1-b2 domains, Nrp1 binds and potentiates the proangiogenic activity of VEGF-A165, which contains the heparin-binding peptide encoded by exon 7 [13]. In addition, Nrp1 acts as the ligand-binding subunit of the receptor complex for the antiangiogenic SEMA3A [14–20], whose sema and immunoglobulin-basic domains, respectively, bind the a1-a2 and b1-b2 domains of Nrp1 [21]. The MAM/c domain instead mediates the SEMA3A-elicited Nrp1 oligomerization that is required for SEMA3A biological activity [21]. Interestingly, the short cytoplasmic domain of Nrp1 is not required for SEMA3A signaling in neurons [22]. In addition, the extracellular b1-b2 domains of Nrp1 mediate heterophilic cell adhesion independently of VEGF-A165 and SEMA3A [11].

*Nrp1* null mice display an embryonic lethal phenotype, characterized by dramatic vascular defects ascribed to impaired angiogenic sprouting [23], branching [24], or arterialization [25] that is significantly more severe than and/or qualitatively different from that of mice lacking either VEGF-A164 (*VEGF^120/120^* mice) [26] or Sema3A [16]. Indeed, although *Nrp1* ^–/–^ embryos die in utero by 13.5 days postcopulation [23], *VEGF^120/120^* pups are recovered at birth at a normal Mendelian frequency [27]. Moreover, a major feature of *Nrp1* null mutants, i.e., the severe impairment of neural tube vascularization [23], is not phenocopied by *VEGF^120/120^* mouse embryos [26]. In addition, differently from *Nrp1* null mice [23,24], the vascular phenotype of *Sema3a* null mice is significantly influenced by the genetic background [16,28–30]. These findings suggest that the vascular defects caused by the loss of *Nrp1* could be due to a VEGF-A164/SEMA3A-independent function of Nrp1 in vascular cells, such as adhesion to the ECM [31,32]. However, how Nrp1 regulates integrin-dependent EC linkages to the surrounding matrix is still obscure. Here, we shed light on the molecular mechanisms by which Nrp1, via its short cytoplasmic domain and independently of VEGF-A165 and SEMA3A, specifically controls a biological function that is crucial for vascular development [3], namely, α5β1-mediated EC adhesion to FN*.*

## Results

### Nrp1 Specifically Promotes EC Adhesion to FN and FN Fibrillogenesis

**Figure 1:**
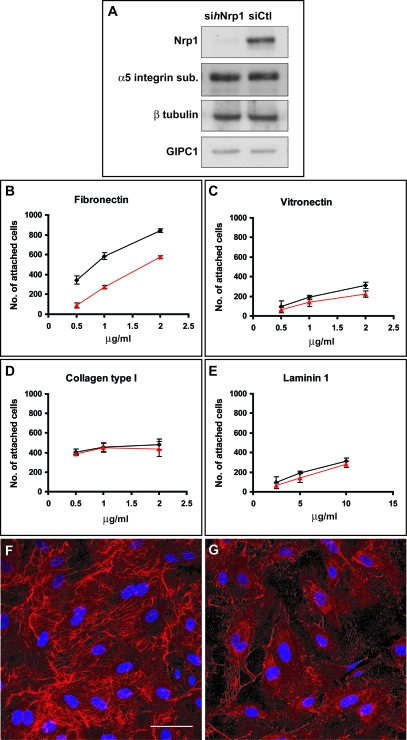
Nrp1 Is Required for EC Adhesion to FN and FN Fibrillogenesis (A) Western blot analysis of protein expression in human ECs silenced for Nrp1 (si*h*Nrp1) or transfected with control siRNA (siCtl). (B–E) Comparison between siCtl (black lines) and si*h*Nrp1 (red lines) transfected ECs adhering to different ECM proteins, i.e., FN (B), VT (C), COLL-I (D), and LN (E). (F–G) Confocal scanning microscopy analysis of endogenous FN fibrils in siCtl (F) or si*h*Nrp1 (G) transfected ECs. DAPI was used to stain nuclei. White bar in (F) corresponds to 50 μm.

To understand the mechanisms by which Nrp1 modulates EC adhesion to different ECM proteins, we silenced the expression of Nrp1 in human umbilical artery ECs by RNA interference (RNAi). Parenthetically, Nrp1 has been found to be expressed at higher levels in arteries than in veins [33]. Endothelial cells were transfected twice with either a pool of three different small interfering RNAs (siRNAs) targeting human Nrp1 (si*h*Nrp1) or control nontargeting siRNA (siCtl). Twenty-four hours after the second transfection, Western blot analysis revealed that, in comparison with control cells, Nrp1 protein, but neither β-tubulin nor the Nrp1 interactor GAIP interacting protein C terminus, member 1 (GIPC1), was successfully silenced in si*h*Nrp1 ECs (Figure 1A).

Next, we investigated the effect of Nrp1 silencing on EC adhesion to different ECM proteins. Fibronectin, vitronectin (VN), and type I collagen (COLL-I) are typical constituents of the provisional angiogenic ECM [1,3], whereas laminin (LN) isoforms are major components of the vascular basement membrane surrounding both immature and mature blood vessels [34]. Short-term (15 min) adhesion assays showed that loss of Nrp1 greatly reduced EC adhesion to FN but not to VN, COLL-I, or LN (Figure 1B–E), suggesting that positive modulation of cell adhesion by Nrp1 is not a general phenomenon [31] but rather a function restricted to specific ECM proteins, such as FN.

Because FN polymerization by ECs has been suggested to participate in vascular morphogenesis [3], we next examined the role of Nrp1 in the fibrillogenesis of endogenous FN. During FN matrix assembly, current models envisage the binding of sFN to surface integrins, thus causing the conversion of FN to a conformational form that favors fibril formation through interactions with other integrin-bound FN dimers [3]. Endothelial cells were cultured in a medium containing FN-depleted fetal calf serum, and accumulation of endogenous FN into fibrils was then detected by confocal immunofluorescence analysis. In comparison with control cells, si*h*Nrp1 ECs were impaired in their ability to incorporate endogenous sFN into a dense fibrillar network 3 h after plating (Figure 1F and 1G). Time-course real-time reverse transcription PCR (RT-PCR) and Western blot analyses revealed that the endogenous FN fibrillogenesis defect observed in si*h*Nrp1 ECs was not due to a reduction in FN mRNA (Figure S1A) or protein (Figure S1D). Hence, Nrp1 specifically promotes EC adhesion to FN and FN matrix formation.

### The Cytoplasmic Domain of Nrp1 Controls EC Adhesion to FN Independently of VEGF-A165 and SEMA3A

**Figure 2:**
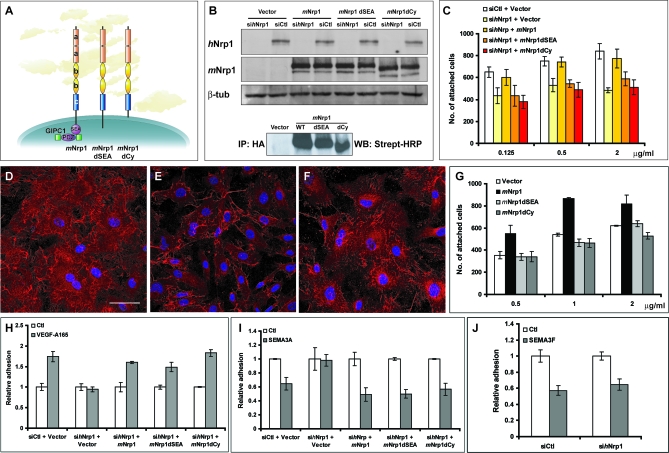
Nrp1 Regulates EC Adhesion to FN and FN Fibrillogenesis via Its Cytoplasmic Domain (A) Schematic representation of *m*Nrp1 full-length and deletion mutants. (B) Western blot analysis of endogenous *h*Nrp1, exogenously transduced *m*Nrp1 constructs, and endogenous β-tubulin expression in ECs transfected with siCtl or si*h*Nrp1 and afterward transduced with PINCO retrovirus carrying *m*Nrp1 constructs. Western blot analysis of biotinylated *m*Nrp1 constructs reveals their correct expression on the surface of ECs. (C) Comparison of wild-type full-length *m*Nrp1 (*m*Nrp1) and *m*Nrp1dSEA and *m*Nrp1dCy efficiency in rescuing the defective adhesion of si*h*Nrp1 ECs to FN. (D–F) Impairment of FN fibrillogenesis in si*h*Nrp1 ECs is rescued by *m*Nrp1 (D) but not by *m*Nrp1dSEA (E) and *m*Nrp1dCy constructs (F). DAPI was used to stain nuclei. White bar in (D) corresponds to 50 μm. (G) *m*Nrp1 overexpression stimulates NIH 3T3 fibroblast adhesion to FN, whereas neither *m*Nrp1dSEA nor *m*Nrp1dCy is active in this respect. (H,I) Silencing *h*Nrp1 completely blocks VEGF-A165-dependent stimulation (H) and SEMA3A-dependent inhibition (I) of human EC adhesion to FN. (J) Silencing *h*Nrp1 in ECs does not impair the *h*Nrp2-dependent inhibition of cell adhesion to FN by SEMA3F.

To start dissecting the mechanisms by which Nrp1 controls the interaction of human ECs with FN, we sought to compare the abilities of full-length and deletion constructs of mouse Nrp1 (*m*Nrp1) to rescue the adhesion and fibrillogenesis defects of si*h*Nrp1 ECs (Figure 2). In particular, we investigated the role played by the extracellular and cytoplasmic moieties of Nrp1. Indeed, the Nrp1 cytodomain, although dispensable for SEMA3A collapsing activity in neurons [22], could signal in cultured ECs [35]. Moreover, the C-terminal SEA sequence of Nrp1 interacts with the PDZ domain of the endocytic adaptor protein GIPC1 [36], whose knockdown during development results in altered arterial branching [37]. Therefore, we transduced si*h*Nrp1 ECs with retroviral vectors carrying the hemagglutinin (HA)-tagged full-length (*m*Nrp1) and deletion mutants of murine Nrp1 (Figure 2A), lacking either the C-terminal SEA amino acids (*m*Nrp1dSEA) or the whole cytoplasmic domain (*m*Nrp1dCy). The si*h*Nrp1 pool did not target any of the *m*Nrp1 constructs, and immunoprecipitation experiments on membrane-biotinylated cell monolayers revealed that all three transmembrane proteins were efficiently exposed on the cell surface (Figure 2B).

In comparison to wild-type *m*Nrp1, both *m*Nrp1dSEA and *m*Nrp1dCy constructs were severely impaired in their abilities to rescue si*h*Nrp1 EC defects in adhesion to FN (Figure 2C) and endogenous FN fibrillogenesis (Figure 2D–F). Accordingly, only *m*Nrp1 overexpression stimulated the adhesion of NIH 3T3 fibroblasts to FN, whereas neither *m*Nrp1dSEA nor *m*Nrp1dCy were active in this respect (Figure 2G). Moreover, *m*Nrp1 overexpression did not promote NIH 3T3 adhesion to VN (Figure S2), further supporting the concept that Nrp1 behaves as a substrate-specific enhancer of cell adhesion. Hence, it appears that the cytoplasmic domain of Nrp1, in particular its SEA motif, which interacts with the endocytic adaptor GIPC1 [36], is required for Nrp1 stimulation of EC spreading on FN and polymerization of endogenous FN.

Opposing autocrine loops of VEGF-A [38–41] and SEMA3A [16,19,42,43] have been found in ECs both in vitro and in vivo. Therefore, we investigated whether the SEA motif and the full cytoplasmic domain of Nrp1 could be required for the modulation of EC adhesion to FN by VEGF-A165 and SEMA3A. Consistent with previous observations [16,18,20,44], silencing Nrp1 completely blocked VEGF-A165-dependent stimulation (Figure 2H) and SEMA3A-dependent inhibition (Figure 2I) of human EC adhesion to FN. As expected, inhibition of cell adhesion to FN by SEMA3F, which signals through Nrp2 [20,21], was not affected by Nrp1 knockdown (Figure 2J). Moreover, similarly to what was observed for SEMA3A in neurons [22], we found that the cytoplasmic domain of Nrp1 is entirely dispensable for both VEGF-A165 (Figure 2H) and SEMA3A (Figure 2I) activity on EC adhesion to FN, because all three *m*Nrp1 constructs rescued si*h*Nrp1 EC response to these factors with a similar efficiency. Thus, the Nrp1 SEA motif and cytodomain are required for Nrp1 modulation of EC adhesion to FN and sFN incorporation into fibrils but not for Nrp1 activity as a VEGF-A165 and SEMA3A coreceptor.

### Nrp1 Regulation of Cell Adhesion Depends on α5β1 Integrin

**Figure 3:**
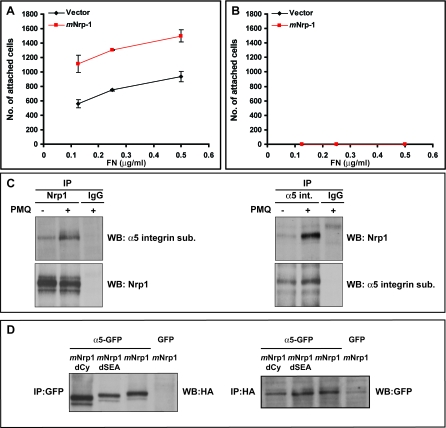
Nrp1 Regulation of Cell Adhesion to FN Requires α5β1 Integrin (A,B) CHO cells expressing (CHO B2α27) (A) or lacking (CHO B2) (B) the α5 integrin subunit were transfected with *m*Nrp1 and allowed to adhere on FN. Overexpression of *m*Nrp1 stimulated CHO cell adhesion to FN in the presence but not in the absence of α5β1 integrin. (C) Immunoprecipitation of endogenous *h*Nrp1 and α5β1 integrin from ECs preincubated for 10 min either in the absence or in the presence of 0.6 μM PMQ, followed, respectively, by Western blotting with anti-α5-integrin Ab and anti-Nrp1 Ab. In ECs, Nrp1 associates with α5β1 integrin, and the recycling inhibitor PMQ increases the stoichiometry of their interaction. (D) NIH 3T3 fibroblasts were cotransfected with the GFP-tagged α5 integrin subunit or GFP alone together with an HA-tagged version of *m*Nrp1 full-length (*m*Nrp1) or *m*Nrp1dSEA or *m*Nrp1dCy deletion constructs. α5-GFP, but not GFP alone, coimmunoprecipitates with all HA-tagged *m*Nrp1 constructs.

α5β1 Integrin is the main FN receptor in ECs [1,3], and by transmitting the actin-dependent tension to sFN, it triggers FN fibrillogenesis [45]. To elucidate whether Nrp1 stimulation of cell adhesion to FN was directly mediated by Nrp1 or was dependent on α5β1 integrin, CHO cells lacking (CHO B2) or expressing (CHO B2α27) the α5 integrin subunit were transfected with *m*Nrp1 and allowed to adhere to FN. Overexpression of *m*Nrp1 stimulated CHO cell adhesion to FN in the presence (CHO B2α27; Figure 3A) but not in the absence (CHO B2; Figure 3B) of α5β1 integrin. Therefore, Nrp1′s proadhesive activity on FN is nonautonomous and mediated by α5β1 integrin.

We then examined whether in ECs Nrp1 could interact physically with α5β1 integrin. Lysates from ECs adhering on endogenous ECM were immunoprecipitated with an antibody (Ab) recognizing the FN receptor α5β1 and then blotted with anti-Nrp1 Ab. Nrp1 coimmunoprecipitated with α5β1, and blotting Nrp1 immunoprecipitates with anti-α5β1-integrin Ab further confirmed the association between endogenous *h*Nrp1 and α5β1 integrin in ECs (Figure 3C). To better understand whether the Nrp1 cytoplasmic domain was required for the interaction with α5β1 integrin, lysates of NIH 3T3 fibroblasts overexpressing HA-tagged full-length or deletion constructs of *m*Nrp1 and green fluorescent protein (GFP)-tagged α5 integrin subunit (α5-GFP) [46] were immunoprecipitated with anti-GFP Ab and then blotted with anti-HA Ab (Figure 3D). We found that both the C-terminal SEA and the cytoplasmic domain of Nrp1 were fully dispensable for its interaction with α5β1 integrin.

**Figure 4:**
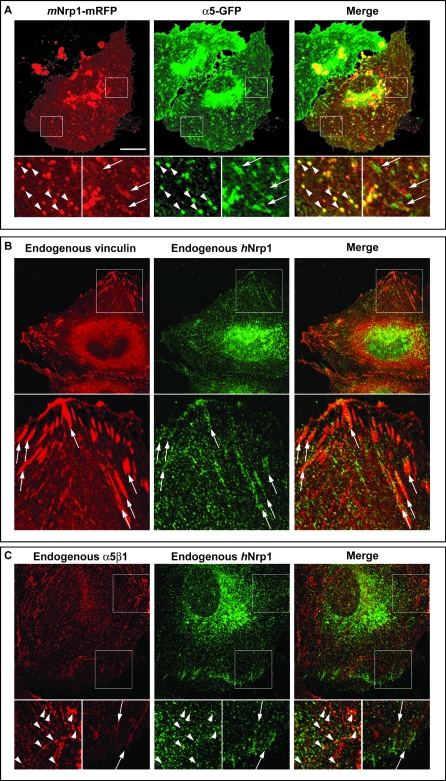
Nrp1 Colocalizes with α5β1 Integrin at Adhesion Sites and Trafficking Vesicles Fluorescent confocal microscopy analysis of untransfected or transfected ECs allowed to adhere for 3 h on FN. (A) In transfected ECs, *m*Nrp1-mRFP (red) and α5-GFP (green) are in close association at adhesion sites (arrows) and colocalize in intracellular vesicles (arrowheads), as visible in merging (right panels). (B) Immunofluorescence analysis reveals that both endogenous *h*Nrp1 and vinculin are enriched in adhesion sites of human ECs (arrows). (C) Similar to what is observed with fluorescent protein-tagged constructs, immunofluorescence analysis showed that endogenous *h*Nrp1 and α5β1 integrin closely associate in adhesion sites (arrows) and colocalize in intracellular vesicles (arrowheads). Lower panels are magnifications of the indicated boxed areas. White bar in (A) corresponds to 25 μm.

To understand the spatial and functional relationships between Nrp1 and α5β1 integrin in ECs, we first generated a monomeric red fluorescent protein (mRFP)-tagged *m*Nrp1 construct (*m*Nrp1-mRFP) that was then cotransfected with α5-GFP in ECs. Fluorescent confocal microscopy showed that at the plasma membrane of ECs adhering on FN *m*Nrp1-mRFP was enriched in close proximity to, or even tightly intermingled with, α5-GFP-containing adhesion sites (Figure 4A, arrows). Moreover, *m*Nrp1-mRFP and α5-GFP fully colocalized in intracellular vesicles (Figure 4A, arrowheads). Notably, immunofluorescence analysis of endogenous endothelial proteins confirmed the spatial links between *h*Nrp1 and vinculin (Figure 4B) or α5β1 integrin (Figure 4C) at either adhesion sites (Figure 4B and 4C, arrows) or vesicular structures located in their proximity (Figure 4C, arrowheads). The observation that Nrp1 and α5β1 colocalization was particularly apparent in intracellular vesicles indicated that these two molecules may associate at or near the point of endocytosis and that they may be internalized as a complex, which is then subsequently disassembled upon recycling to the plasma membrane. We have previously found that endosomal integrin complexes can be preserved by treating the cell with primaquine (PMQ), a receptor recycling inhibitor, prior to lysis [47]. Therefore, we immunoprecipitated α5β1 integrin or Nrp1 from cells that had been treated with PMQ for 10 min and probed for the presence of the α5β1/Nrp1 complex by Western blotting. Pretreatment of the cells with PMQ greatly increased the coprecipitation of α5β1 integrin with Nrp1 and vice versa (Figure 3C), indicating the likelihood that this complex is more stable in endosomes than at the plasma membrane.

**Figure 5:**
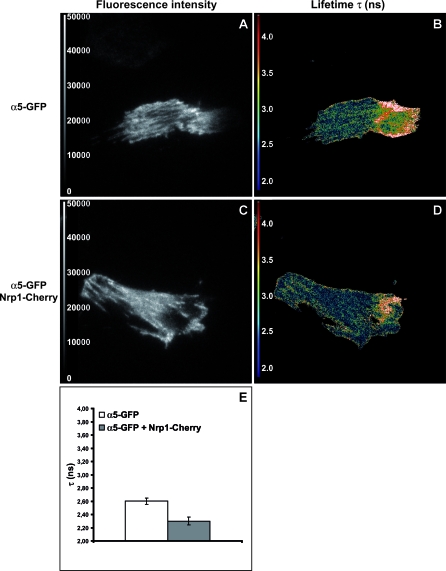
TIRF/FLIM Analysis of the Fluorescence Resonance Energy Transfer between α5-GFP and *m*Nrp1-Cherry NIH 3T3 fibroblasts were transfected with either α5-GFP alone (A,B) or cotransfected with α5-GFP and *m*Nrp1-Cherry (C,D) and plated onto FN-coated glass-bottom dishes. (A,C) Fluorescent intensity images of α5-GFP excited in TIRF with a 473-nm laser show the expected α5-GFP localization in adhesion sites. (B,D) Pseudocolor images of the spatial distribution of donor (α5-GFP) fluorescence lifetimes τ (measured in nanoseconds) were obtained by frequency-domain FLIM analysis of TIRF fluorescence images shown in (A) and (C). (E) In comparison with cells transfected with α5-GFP alone (*n* = 7), the donor lifetime τ is decreased (from 2.6 ± 0.05 to 2.3 ± 0.06 ns) in adhesion sites of cells expressing both α5-GFP and *m*Nrp1-Cherry (*n* = 7; *P* = 0.00000005).

To further characterize the interaction between Nrp1 and α5β1 integrin, we measured fluorescence resonance energy transfer (FRET) in live NIH 3T3 cells transfected with α5-GFP alone or cotransfected with α5-GFP and *m*Nrp1 tagged with the fluorescent protein Cherry, an improved version of mRFP (*m*Nrp1-Cherry). Total internal reflection fluorescence (TIRF) illumination [48] was used to selectively excite α5-GFP at the basal cell plasma membrane where ECM adhesions lie. Fluorescence resonance energy transfer was measured by fluorescence lifetime imaging microscopy (FLIM) [49] and was read out as a decrease in donor (GFP) fluorescence lifetime. We found that the α5-GFP fluorescence lifetime was significantly reduced in cells that coexpressed *m*Nrp1-Cherry, indicating that FRET, and thus a close physical interaction, was occurring between α5β1 and Nrp1 at adhesion sites with an 11.5% FRET efficiency (Figure 5).

Taken together, these data indicate that in living cells Nrp1 physically associates with α5β1 at or near sites of cell–ECM contact and that this interaction is likely maintained following internalization of the complex.

### Nrp1 Controls the Traffic of Active α5β1 Integrin

**Figure 6:**
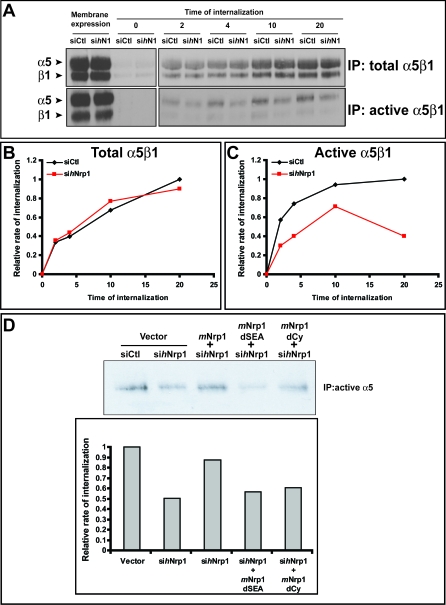
Nrp1 Regulates the Traffic of Active α5β1 Integrin in ECs (A) Time-course assays reveal an impairment of active but not total α5β1 integrin internalization in ECs silenced for *h*Nrp1 (si*h*N1) in comparison with cells transfected with control siRNA (siCtl). (B,C) Relative quantifications of time-course internalization assays shown in (A) of total (B) and active (C) α5β1 integrin are depicted. (D) Wild-type *m*Nrp1, but neither *m*Nrp1dSEA nor *m*Nrp1dCy deletion constructs, was able to rescue the early (4 min) internalization defects of active α5β1 integrin in si*h*Nrp1 ECs as quantified in the lower histogram.

The efficiency of cell adhesion and spreading on ECM is generally thought to be proportional to the amount of either active or total (i.e., active and inactive) integrin at the cell surface [1,6]. We found that lack of Nrp1 did not alter the global amount of either total (Figure 1A), as already reported [31], or active α5β1 integrin, as recognized by the mouse monoclonal Ab (mAb) SNAKA51 [45] (Figure S3). Then, we analyzed whether Nrp1 could influence the amount of α5β1 integrin on the endothelial surface. Biotinylation experiments revealed that knocking down human Nrp1 did not diminish the surface levels of either total or active α5β1 integrin in si*h*Nrp1 ECs (Figure 6A), thus suggesting that a mechanism alternative to the control of integrin conformation should be responsible for Nrp1-dependent activation of α5β1 integrin function in ECs.

On the basis of our observations that Nrp1 and α5β1 integrin colocalize in intracellular vesicles (Figure 4A and 4C) and that inhibition of recycling by PMQ increased the association of Nrp1 with α5β1 integrin (Figure 3C), we decided to monitor the effect of Nrp1 knockdown on the internalization of total and active surface α5β1 integrin. Endothelial cells were surface-labeled with cleavable biotin at 4 °C and incubated at 37 °C for different times to allow internalization, and then biotin remaining on cell-surface proteins was cleaved at 4 °C [50]. Integrin internalization was quantified by immunoprecipitation of either total (Figure 6A and 6B) or active (Figure 6A and 6C) α5β1 integrin, followed by Western blot analysis with streptavidin. Notably, although endocytosis of the cell-surface pool of total α5β1 integrin (i.e., active plus inactive heterodimers) was not detectably altered in si*h*Nrp1 cells (Figure 6A and 6B), knockdown of Nrp1 markedly reduced the quantity of active (SNAKA51-positive) α5β1 heterodimers internalized by ECs (Figure 6A and 6C). Taken together, these data indicate that on the cell surface Nrp1 interacts with active α5β1 heterodimers at adhesion sites (Figure 4A and 4C, arrows) and acts to promote their internalization and localization to intracellular vesicles (Figure 4A and 4C, arrowheads).

**Figure 7:**
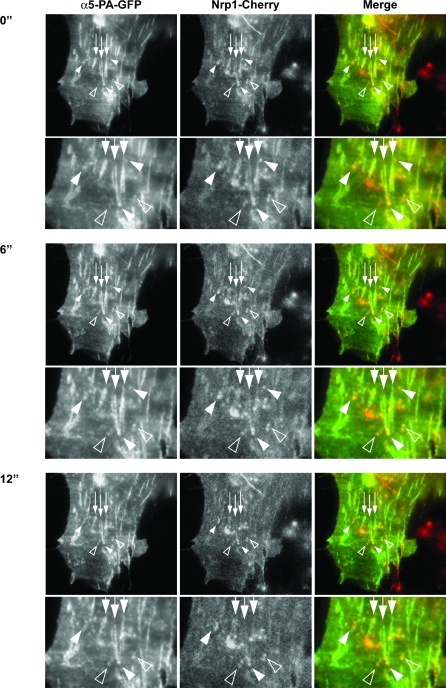
Rapid Turnover of α5β1 Integrin from Adhesion Sites into Nrp1-Positive Vesicles A representative NIH 3T3 cell was cotransfected with *m*Nrp1-Cherry and α5-PA-GFP, photoactivated in TIRF, and observed in time-lapse epifluorescence microscopy. After photoactivation, α5-PA-GFP integrin (left panels) was found in elongated adhesive structures (i.e., fibrillar adhesions; arrows), where it colocalized with *m*Nrp1-Cherry (middle panels), as evident in merged images as well (right panels). α5-PA-GFP integrin is also present in nascent vesicles near adhesive structures (arrowheads). Over time GFP fluorescence intensity (left panels) is constant in some vesicles (solid arrowheads), while it increases in others (empty arrowheads), that become progressively enriched in α5-PA-GFP integrin deriving from membrane adhesion sites. *m*Nrp1-Cherry (middle panels) is also present in the same vesicles (solid and empty arrowheads), as visible in merged images (right panels). At each time point, lower panels are magnifications of the corresponding upper panels (see also Video S1).

To visualize the internalization and postendocytic trafficking of the α5β1/Nrp1 complex, we deployed the photoactivatable (PA) α5-GFP (α5-PA-GFP) probe that we had previously used to monitor α5β1 trafficking in human ovarian carcinoma A2780 cells [51]. However, the multitude of fluorescent vesicles travelling to and from the cell surface made it difficult to track the progress of individual α5β1 integrin transport vesicles. Therefore, we used TIRF to restrict the plane of activating fluorescence, such that only α5-PA-GFP present at or near the cell surface became photoactivated. Then we tracked the movement of this photoactivated fraction of α5β1 integrin using time-lapse epifluorecence microscopy. With this novel technique, α5β1 integrin was photoactivated almost exclusively at adhesion sites (mostly fibrillar adhesions), where it colocalized with *m*Nrp1-Cherry (Figure 7, arrows). Photoactivated α5β1 was then rapidly (<6 s) internalized and cotransported with *m*Nrp1-Cherry in small endocytic vesicles (Figure 7, empty arrowheads, and Video S1) that moved away from the fibrillar adhesions. In addition, we found that α5β1 integrin turnover in ECM adhesions was unexpectedly very rapid (Figure S4 and Video S2), with the α5-PA-GFP signal leaving the adhesive sites, accumulating in vesicles, and disappearing by ∼45 s after photoactivation in approximately 50% of the adhesion sites and by ∼115 s in the remaining ones (Video S2).

**Figure 8:**
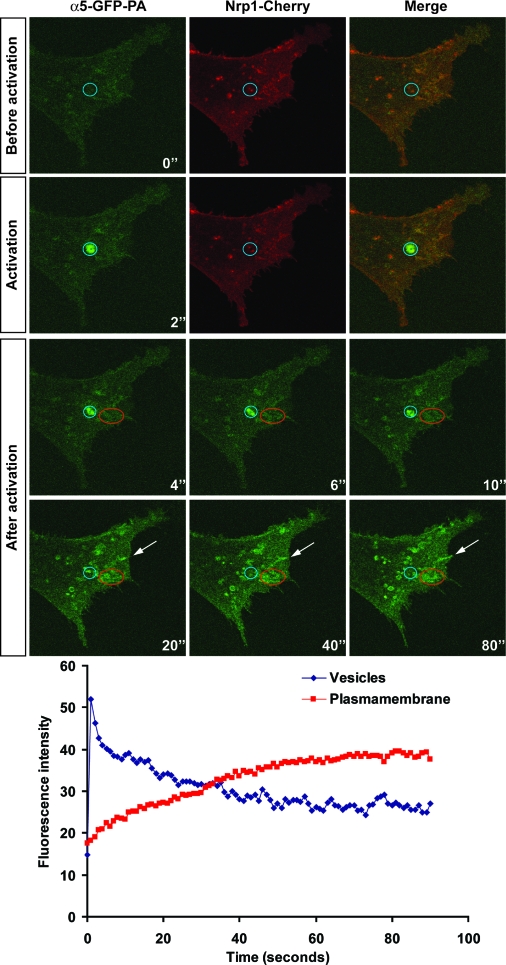
Upon Photoactivation in *m*Nrp1-Cherry-Positive Vesicles, α5-PA-GFP Recycles Back to Membrane Adhesions NIH 3T3 cells were cotransfected with *m*Nrp1-Cherry and α5-PA-GFP. α5-PA-GFP was then locally photoactivated in vesicles containing *m*Nrp1-Cherry (blue circle) and followed by time-lapse confocal microscopy. Fluorescence intensity was measured over time in Nrp1-positive vesicles (blue circle) and at the plasma membrane (red circle). The time-lapse plot (lower panel) shows that the α5-PA-GFP fluorescence intensity decreases in the vesicles, while it increases at the plasma membrane. This means that α5-PA-GFP integrin is recycled from Nrp1-containing vesicles to the plasma membrane, where it appears to be enriched in what looks like adhesive structures (red circle and white arrows) (see also Video S3).

Having established that α5β1 integrin and Nrp1 are cointernalized at fibrillar adhesions, we wished to determine whether the integrin was then recycled from Nrp1-positive vesicles back to the plasma membrane. To address this, we aimed a pulse of 405-nm laser light at a “single point” corresponding to Nrp1-positive vesicles, leading to the immediate photoactivation of α5-PA-GFP integrin largely within the confines of these structures (Figure 8 and Video S3). During the following 80 s, fluorescence was lost from the photoactivated vesicle, and this was accompanied by a corresponding increase in integrin fluorescence at peripheral elongated structures that look like adhesion sites (Figure 8, red circle and white arrows). On the contrary, when the activating laser was aimed at a cell region devoid of Nrp1-positive vesicles, little or no photoactivation occurred (Figure S5 and Video S4), indicating that the α5-PA-GFP fluorescence detected in Figure 8 was indeed at *m*Nrp1-Cherry vesicles and not at the plasma membrane above and below them.

Taken together, these data indicate that α5β1 integrin and Nrp1 are cointernalized into intracellular vesicles, which are then rapidly returned or recycled to the plasma membrane. Interestingly, both the internalization and the recycling of Nrp1-associated α5β1 integrin occur at the site of adhesion to the ECM.

### Cytoplasmic Domain of Nrp1 Elicits the Endocytosis of Active α5β1 Integrin via GIPC1 in ECs

**Figure 9:**
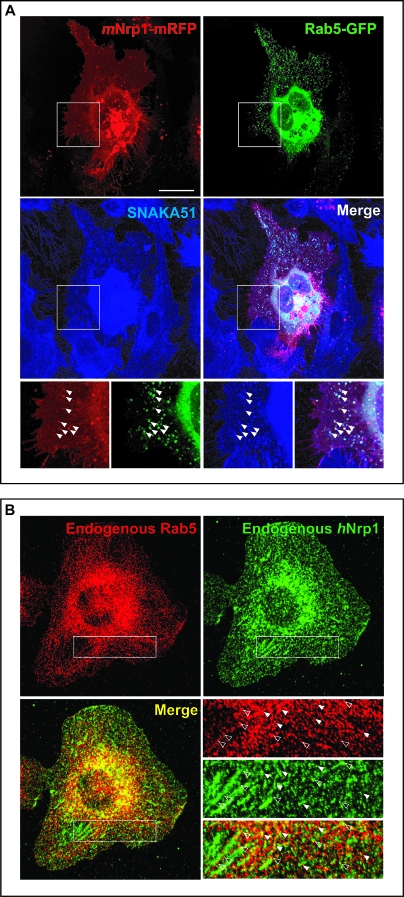
Nrp1 and Active α5β1 Integrin Localize into Rab5-Positive Early Endosomes (A) Fluorescent confocal microscopy analysis of ECs transfected with *m*Nrp1-mRFP and Rab5-GFP and then incubated with the anti-active-α5β1 mAb (SNAKA51). *m*Nrp1-mRFP and active α5β1 colocalize into Rab5-GFP-positive early endosomes as shown in merging (arrowheads). Lower panels are magnifications of the boxed areas shown in the upper panels. (B) Immunofluorescent confocal microscopy analysis of endogenous *h*Nrp1 and Rab5 localization in ECs. As described previously, *h*Nrp1 is concentrated in elongated adhesion sites and in vesicular structures. Endogenous Rab5 colocalizes with *h*Nrp1 into early endosomes (solid and empty arrowheads), many of which are located near adhesion sites (empty arrowheads). Lower-right panels are magnifications of the boxed areas shown in the other panels. White bar in (A) corresponds to 25 μm.

In the eukaryotic early endocytic pathway, the small GTPase Rab5 is a rate-limiting component that regulates the entry of cargoes from the plasma membrane into the early endosome [52]. Hence, we analyzed the early endocytic steps of active α5β1 integrin in ECs cotransfected with *m*Nrp1-mRFP and Rab5-GFP, which were incubated with the α5β1 integrin activation reporter mAb SNAKA51 for 30 min at 4 °C and then at 37 °C for different time points. Fluorescent confocal analysis indicated that, after 1–3 min of internalization at 37 °C, Nrp1 and active α5β1 integrin colocalized in early Rab5-positive vesicles near the EC plasma membrane (Figure 9A). Accordingly, immunofluorescence analysis of endogenous endothelial proteins confirmed that *h*Nrp1 and Rab5 colocalized in vesicles, many of which were located near adhesion sites (Figure 9B, empty arrowheads), further supporting the view that Nrp1 can induce α5β1-mediated adhesion by promoting the preferential internalization of its active conformation into Rab5-positive early endosomes and the ensuing recycling to newly forming cell–ECM contacts.

Next, to characterize the molecular mechanisms by which Nrp1 regulates the traffic of active α5β1 integrin, we evaluated the abilities of *m*Nrp1 full-length and mutant constructs to rescue the integrin internalization defects that we observed in si*h*Nrp1 ECs. Remarkably, only wild-type *m*Nrp1, but neither *m*Nrp1dSEA nor *m*Nrp1dCy construct, was able to rescue the si*h*Nrp1 EC defects in the endocytosis of active α5β1 integrin (Figure 6D). Therefore, in ECs the SEA motif of Nrp1, which binds the endocytic adaptor GIPC1 [36], is mandatory for Nrp1 stimulation of cell adhesion to FN (Figure 2C), endogenous FN fibrillogenesis (Figure 2D–F), and active α5β1 integrin endocytosis (Figure 6D).

**Figure 10:**
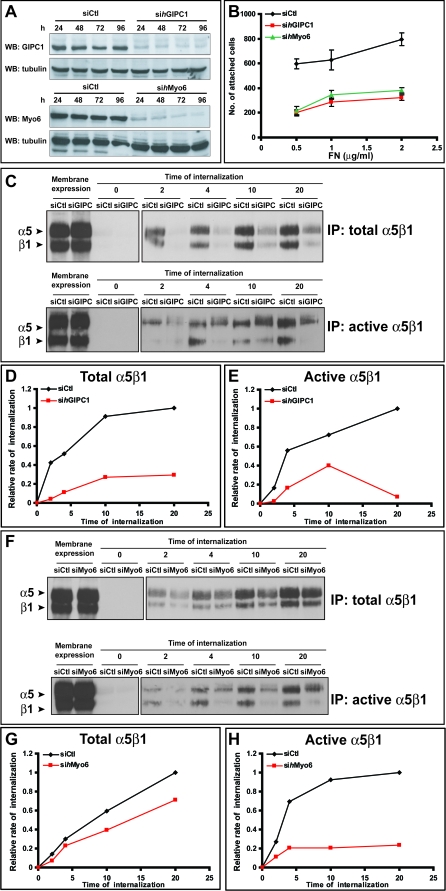
In ECs GIPC1 and Myo6 Regulate α5β1 Integrin Traffic and Function (A) Western blot analysis of protein expression in ECs silenced for human GIPC1 (si*h*GIPC1) or Myo6 (si*h*Myo6) or transfected with control siRNA (siCtl) reveals an efficient silencing of GIPC1 or Myo6 at 96 h after the second oligofection. (B) Comparison between siCtl (black) and either si*h*GIPC1 (red) or si*h*Myo6 (green) transfected ECs adhering to FN. (C) Time-course analysis reveals an impairment of both total and active α5β1 integrin internalization in ECs silenced for *h*GIPC1 in comparison with control cells (siCtl). (D,E) Relative quantification of time-lapse endocytosis assay (shown in (C)) of total (D) or active (E) α5β1 integrin in ECs silenced for *h*GIPC1. (F) Time-course analysis reveals a significant impairment of active but not total α5β1 integrin internalization in ECs silenced for *h*Myo6 in comparison with control cells (siCtl). (G,H) Relative quantification of time-course endocytosis assay (shown in (F)) of total (G) or active (H) α5β1 integrin in ECs silenced for *h*Myo6.

The N-terminal portion of GIPC1 mediates its oligomerization, whereas its central PDZ domain can bind the C-terminal consensus S/T-X-Φ sequence of Nrp1 [36], the α5 integrin subunit [53], and the Rab5/Rab21 interactor protein APPL1 [54,55]. Thus, we theorized that as a result GIPC1 could support the Rab5-dependent early internalization of α5β1 integrin. To test this hypothesis, we silenced the expression of GIPC1 in human umbilical artery ECs by RNAi and examined its effect on α5β1 integrin traffic. Western blot analysis showed that, 96 h after the second transfection, GIPC1 protein, but not β-tubulin, was successfully silenced in si*h*GIPC1 ECs in comparison with control cells (Figure 10A). Knockdown of GIPC1 in ECs dramatically reduced the amount of internalized total (Figure 10C and 10D) and active (Figure 10C and 10E) α5β1 integrin by ∼70% throughout the whole internalization assay, suggesting that indeed the interaction of α5β1 integrin with GIPC1 is crucial for the endocytosis and the proper functioning of this integrin. Accordingly, short-term adhesion assays showed that, in comparison with control cells, si*h*GIPC1 ECs adhered poorly to FN (Figure 10B) and much less efficiently assembled endogenous sFN into a fibrillar network (Figure S1H) in comparison with cells transfected with siCtl (Figure S1G). The latter defect was not due to a reduction in FN mRNA or protein levels as demonstrated by real-time RT-PCR (Figure S1B) and Western blotting (Figure S1E). Hence, within Nrp1 the extracellular domain mediates the association with α5β1 integrin, and the C-terminal SEA sequence allows the binding to the endocytic adaptor GIPC1 that stimulates the internalization and traffic of active α5β1 integrin, finally promoting EC adhesion to FN and FN fibrillogenesis.

### GIPC1 Interacting Motor Myo6 Promotes Active α5β1 Endocytosis, EC Adhesion to FN, and *FN1* Gene Transcription

Because the C terminus of GIPC1 binds to the minus-end-directed motor myosin VI (Myo6) that has also been involved in endocytosis [56], we considered the hypothesis that Myo6 could cooperate with GIPC1 in promoting α5β1 integrin internalization. Interestingly, RNAi-mediated knockdown of Myo6 in human umbilical artery ECs (Figure 10A) resulted in a significant (∼70%) impairment of active α5β1 integrin internalization (Figure 10F and 10H), whereas the total integrin pool was only mildly affected (∼25%; Figure 10F and 10G). These data, together with the fact that si*h*Myo6 EC adhesion to FN was severely hampered (Figure 10B), indicate that Myo6 cooperates with GIPC1 in the regulation of active α5β1 integrin endocytosis.

Similarly to what we noticed after Nrp1 and GIPC1 knockdown, ECs in which Myo6 was silenced did not efficiently assemble an endogenous FN fibrillar network (Figure S1I) in comparison with cells transfected with siCtl (Figure S1G). However, differently from what we observed in si*h*Nrp1 and si*h*GIPC1 ECs, the endogenous FN fibrillogenesis defect seen in si*h*Myo6 ECs was due to an inhibition of *FN1* gene transcription mRNA (Figure S1C), which associated to a significant reduction of FN protein levels as well (Figure S1F). Indeed, in addition to its role in cytoplasmic transporting and anchoring, Myo6 is also present in the nucleus, where it promotes the RNA-polymerase-II-dependent transcription of active genes [57]. Here we identify the *FN1* gene as a new Myo6 transcriptional target and downstream effector that can bolster EC adhesion and motility.

## Discussion

Defects of developing blood vessels caused by *Nrp1* gene knockdown in mice [23,24] are different from vascular malformations displayed by mice lacking either SEMA3A [16] or VEGF-A165 (*Vegf-a^120/120^* mice) [26]. Furthermore, it has been recently reported that Nrp1 is required for EC responses to both VEGF-A165 and VEGF-A121 isoforms, the latter being incapable of binding Nrp1 on the EC surface [58,59]. Therefore, it is conceivable that the vascular abnormalities of *Nrp1^–/–^* mice could be due at least in part to the disruption of a VEGF-A165/SEMA3A-independent Nrp1 function. α5β1 Integrin and its ligand FN are key players in vascular development [3]. The data reported here support a model in which Nrp1, through its cytoplasmic domain and independently of its activity as a SEMA3A and VEGF-A165 coreceptor, stimulates GIPC1/Myo6-dependent endocytosis and traffic of active α5β1 integrin, thus promoting EC adhesion to FN and FN fibrillogenesis.

In rescue experiments, where we reintroduced full-length and mutant murine Nrp1 constructs in human ECs in which endogenous *h*Nrp1 was simultaneously knocked down by RNAi, we showed that EC adhesion to FN and polymerization of endogenous sFN into fibrils depend on the cytoplasmic domain of Nrp1, the C-terminal SEA motif representing the minimal sequence required to exert these functions. Importantly, as already shown for SEMA3A-elicited growth cone collapse in neurons [22], we found that the cytoplasmic domain of Nrp1 is instead dispensable for VEGF-A165 stimulation and SEMA3A inhibition of EC adhesion to FN. Moreover, by using two CHO cell clones differing in the expression of α5β1 integrin, we demonstrated that Nrp1 alone does not directly mediate adhesion to FN and that it requires α5β1 integrin. Therefore, we conclude that in ECs, independently of VEGF-A165 and SEMA3A, Nrp1 stimulates α5β1-mediated adhesion to FN and endogenous FN fibrillogenesis via its cytoplasmic SEA motif [36]. This motif, similar to the C-terminal SDA sequence of the α5 integrin subunit [53], selectively and specifically binds the PDZ domain of the homomultimeric endocytic adaptor GIPC1.

It is known that conformational activation of cell-surface integrins supports cell adhesion and spreading, whereas transition of integrins toward an inactive bent conformation causes cell de-adhesion and rounding up [1,60,61]. However, we observed that lack of Nrp1 does not result in the reduction of either active or total α5β1 integrin either at the cell surface or intracellularly. Rather, by combining biochemical analysis with conventional and TIRF/FLIM confocal microscopy, we found that at the plasma membrane Nrp1 is tightly associated with adhesion sites, where it physically interacts with α5β1 integrin. The complex formed between active α5β1 integrin and Nrp1 is then rapidly internalized into Rab5-positive endosomes in an Nrp1-dependent fashion. Interestingly, the integrin is then returned to the plasma membrane from Nrp1-containing vesicles, and this recycling event appears to be targeted to adhesive structures. In addition, although the extracellular domain of Nrp1 is sufficient for its interaction with α5β1 integrin, the C-terminal GIPC1-binding SEA sequence of Nrp1 is necessary for stimulating EC adhesion to FN. Accordingly, knocking down either GIPC1 or its interacting motor Myo6 results in a significant impairment of active α5β1 integrin endocytosis and EC adhesion to FN.

**Figure 11:**
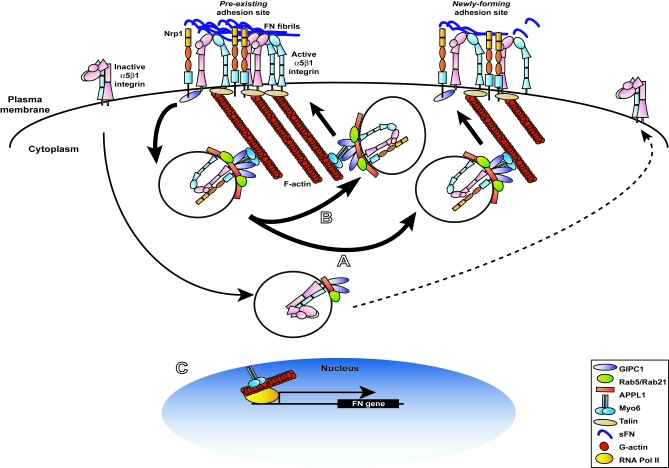
Model for Nrp1 Regulation of α5β1 Integrin Traffic and Function in ECs (A) At adhesive sites of ECs spreading on FN, Nrp1, via its cytoplasmic association with oligomers of the endocytic adaptor GIPC1, promotes the Rab5/Rab21-dependent internalization of active α5β1 integrin. Once endocytosed, active α5β1 is then recycled back from Nrp1-positive vesicles to the cell surface, thus favoring the dynamic rehandling of newly forming adhesion sites. GIPC1 oligomers could facilitate the association of the α5 integrin subunit with the Bin-Amphiphysin-Rvs (BAR) protein and Rab5/Rab21 interactor APPL1. Myo6 associates with and assists GIPC1 in promoting active α5β1 endocytosis and the ensuing postendocytic traffic. (B) Moreover, in adherent cells a steady endo-exocytic flow of (active) α5β1 integrins from and toward existing ECM adhesions could allow cells to rapidly adjust polarity and cell–ECM contacts in response to extracellular stimuli. (C) In addition, Myo6 can translocate to the EC nucleus, where it stimulates the RNA-polymerase-II-dependent transcription of the *FN1* gene.

Taken together, our data indicate that, during EC adhesion and spreading on FN, Nrp1, through its extracellular domain, transiently interacts with active α5β1 integrin at adhesive sites and, via its cytoplasmic association with GIPC1, enhances the early endocytosis and the ensuing recycling of active α5β1 integrin to newly forming adhesion sites (Figure 11A). It is therefore likely that fast cycles of endocytosis from and recycling to ECM adhesions of active α5β1 integrin could allow real-time optimization of adhesion during EC spreading on FN. These conclusions are in line with the recent findings by Ivaska and colleagues [8,9] that found how endocytosis of β1 integrins, in addition to their established role in directional migration [7], regulates cell adhesion and spreading as well. In particular, they reported that class V Rab GTPases (for review, see [52]) Rab21 and Rab5 directly bind to several integrin α subunits, α5 included, by interacting with the conserved membrane proximal region GFFKR, which interestingly has been previously implicated in conformational integrin activation [61]. It is thus conceivable that GIPC1 oligomers could favor α5β1 integrin endocytosis by bridging the α5 integrin subunit and the Rab5/Rab21 interactor APPL1, finally stabilizing the interaction between these small GTPases and α5β1 integrin. This could represent a main functional feature distinguishing α5β1 from other integrin heterodimers not interacting with GIPC1. Finally, the fact that by 2 min after activation α5-PA-GFP disappeared from preexisting adhesion sites into vesicles without a concomitant cell retraction suggests the existence of a steady endo-exocytic flow of (active) α5β1 integrins from and toward existing ECM adhesions as well (Figure 11B). This mechanism could allow adherent cells to be always ready to rapidly exchange integrins among cell–ECM contacts in response to extracellular stimuli. Such a scenario is also compatible with a previous study by Ezratty and colleagues [62] and implies that disassembly of ECM adhesions could depend on an imbalance of endocytosis over recycling.

Our observation that Myo6 siRNA severely impairs EC adhesion to FN and results in a significant reduction in the internalization of active α5β1 integrin suggests that Myo6 cooperates with GIPC1 (Figure 11A and 11B) and is compatible with the notion that Myo6 plays a role in the formation and transport of endocytic vesicles along F-actin microfilaments [56]. The decrease in FN mRNA that we noticed in si*h*Myo6 ECs is likely due to the lack of the transcriptional activity displayed by Myo6 in the nucleus [57] that could depend on a still not fully characterized actin–myosin-based mechanism of transcription [63,64]. Therefore, Myo6 can support EC adhesion and motility by promoting both active α5β1 integrin traffic (Figure 11A and 11B) and *FN1* gene transcription (Figure 11C). Additionally, these findings can have significant implications for the biology of α5β1-expressing human carcinomas [51,65], in which Myo6 can be overexpressed and promote metastatic invasion [66–68].

In conclusion, we propose here that Nrp1, in addition to and independently of its role as coreceptor for VEGF-A165 and SEMA3A, stimulates through its cytoplasmic domain the spreading of ECs on FN by increasing the Rab5/GIPC1/Myo6-dependent internalization of active α5β1 integrin. Nrp1 modulation of α5β1-mediated adhesion can play a causal role in the generation of angiogenesis defects observed in *Nrp1* null mice. We anticipate that signaling pathways controlling Nrp1 expression in ECs could ultimately modulate the activity of α5β1 integrin. In particular, Nrp1 is a major target of the inhibitory Delta-like 4–Notch signaling pathway [69] that negatively regulates the formation of endothelial tip cells [10]. Higher expression of Nrp1 in tip ECs compared with that in stalk ECs of angiogenic sprouts could differentially modulate α5β1 integrin traffic, thus favoring tip cell adhesion and spreading on FN. Finally, both Nrp1 [70] and α5β1 integrin [71,72] are expressed in pericytes and vascular smooth muscle cells, which have been implicated in vascular remodeling by intussusceptive angiogenesis [73]. Further work is needed to assess whether Nrp1 is regulating α5β1 integrin function not only in ECs but also in pericytes and vascular smooth muscle cells.

## Materials and Methods

### Antibodies, recombinant proteins, and growth factors.

Goat polyclonal anti-Nrp1 (C-19) and rabbit polyclonal anti-β-tubulin (H-235) were from Santa Cruz Biotechnology. Mouse monoclonal anti-human-Nrp1 (MAB 3870) was from R&D Systems. Mouse monoclonal anti-FN (MAB88904) and anti-αvβ3-integrin (MAB1976), goat polyclonal anti-α5β1-integrin (AB1950), rabbit polyclonal anti-α5-integrin (AB1928), rabbit polyclonal anti-α2-integrin (AB1936), and anti-α3-integrin (AB1920) were from Chemicon. Mouse monoclonal anti-human-vinculin (V9131) and rabbit polyclonal anti-Rab5 (R4654) were from Sigma-Aldrich. Rat monoclonal anti-HA (3F10) was from Roche. Rabbit polyclonal anti-GFP (A11122) and 4′,6-diamidino-2-phenylindole (DAPI) were from Molecular Probes. Goat polyclonal anti-GIPC1 (ab5951) and rabbit polyclonal anti-Myo6 (ab11096) were from Abcam. Streptavidin–horseradish peroxidase was from Amersham. Mouse monoclonal anti-active-α5-integrin, SNAKA51, was previously described [45].

Human plasma FN was from Tebu-bio. Human plasma vitronectin, Engelbreth-Holm-Swarm murine sarcoma laminin, and calf skin collagen type I were from Sigma-Aldrich. Recombinant human VEGF-A165 was from Invitrogen. Recombinant human SEMA3A and mouse Sema3F were from R&D Systems. Sulfo-NHS-SS-Biotin was from Pierce.

### DNA constructs.

Hemagglutinin-tagged *m*Nrp1 deletion constructs were generated by standard PCR protocols according to the Taq polymerase manufacturer's instructions (Fynnzymes) and using an HA-tagged version of full-length *m*Nrp1 kindly donated by A. Püschel (Westfälische Wilhelms-Universität, Münster, Germany) as template. Cytoplasmic domains and the last three amino acids SEA were deleted using the following oligonucleotide primers: (i) 5′-cgccatggagagggggctgccgttg-3′ (Fw); (ii) 5′-ccaacaggcacagtacag-3′ (Re1) to amplify the *m*Nrp1 deleted of the cytoplasmic domain; (iii) 5′-gtaattactctgtgggttc-3′ (Re2) to amplify the *m*Nrp1 deleted of the three amino acids SEA. The corresponding PCR product was first thymidine–adenine (TA)-cloned into pCR2.1TOPO (Invitrogen) and subsequently subcloned in PINCO retrovirus or pAcGFP-N1 Vector (BD Bioscience) whose GFP coding sequence was previously substituted with the cDNA of mRFP, a kind gift of R. Tsien (University of California, San Diego, CA). α5-GFP and Rab5-GFP constructs were kindly provided, respectively, by A.F. Horwitz (University of Virginia, Charlottesville, VA) and M. Zerial (Max Plank Institute of Molecular Cell Biology and Genetics, Dresden, Germany).The α5-PA-GFP construct was previously described [51].

### siRNA.

The day before oligofection, ECs were seeded in six-well dishes at a concentration of 10 × 10^4^ cells/well. Oligofection of siRNA duplexes was performed according to the manufacturer's protocols. Briefly, human ECs were transfected twice (at 0 and 24 h) with 200 pmol of siCONTROL nontargeting siRNA (as control), siGENOME SMART pools (in the case of *h*GIPC1 and *h*Myo6), or a mix of three (in the case of *h*Nrp1) siRNA oligonucletides (Dharmacon). After 24 h (in the case of *h*Nrp1) or 96 h (in the case of *h*GIPC1 or *h*Myo6) had passed since the second oligofection, ECs were lysed or tested in functional assays. In the case of *h*Nrp1, the single oligonucleotide sequences were: (1) 5′-AAUCAGAGUUUCCAACAUA-3′; (2) 5′-GAAGGAAGGGCGUGUCUUG-3′; (3) 5′-GUGGAUGACAUUAGUAUUA-3′.

### Adhesion assay.

Six-thousand ECs were resuspended in 0.1 ml of EBM-2 (Clonetics) with or without appropriate stimuli (50 ng/ml VEGF-A, 200 ng/ml SEMA3A, and 400 ng/ml SEMA3F) and plated on 96-well microtiter plates (Costar) that were previously coated with ECM proteins at different concentrations and then saturated with 3% bovine serum albumin. After 15 min of incubation at 37 °C, cells were fixed in 8% glutaraldehyde and then stained with 0.1% crystal violet in 20% methanol. Cells were photographed with a QICAM Fast 1394 digital color camera (QImaging) and counted by means of Image-ProPlus 6.2 software (Media Cybernetics). In adhesion assays with NIH 3T3 fibroblasts or CHO cells, Dulbecco's modified Eagle's medium was used.

### Immunoprecipitation and Western blotting.

Endothelial cells were lysed in buffer containing 25 mM Tris-HCl, pH 7.6, 100 mM NaCl, 0.15% Tween-20, 5% glycerol, 0.5 mM ethylene glycol tetraacetic acid (EGTA), and protease inhibitors (50 mg ml^−1^ pepstatin; 50 mg ml^−1^ leupeptin; 10 mg ml^−1^ aprotinin; 2 mM phenylmethanesulfonylfluoride (PMSF); 2 mM MgCl_2_). Cells were lysed in buffer, incubated for 20 min on wet ice, and then centrifuged at 15,000*g*, 20 min, at 4 °C. The total protein amount was determined using the bicinchoninic acid (BCA) protein assay reagent (Pierce). Equivalent amounts (1,200 μg) of protein were immunoprecipitated for 1 h with the antibody of interest, and immune complexes were recovered on Protein G-Sepharose (GE Healthcare). Immunoprecipitates were washed four times with lysis buffer, twice with the same buffer without Tween-20, and then separated by SDS-PAGE. Proteins were then transferred to a Hybond-C extra nitrocellulose membrane (Amersham), probed with antibodies of interest, and detected by an enhanced chemiluminescence technique (PerkinElmer).

### Integrin internalization assay.

Integrin traffic assays were performed as previously described by Roberts et al. [50] with minor modifications. Cells were transferred to ice, washed twice in cold phosphate-buffered saline (PBS), and surface-labeled at 4 °C with 0.2 mg/ml sulfo-NHS-SS-biotin (Pierce) in PBS for 30 min. Labeled cells were washed in cold PBS and transferred to prewarmed EGM-2 at 37 °C. At the indicated times, the medium was aspirated, and dishes were rapidly transferred to ice and washed twice with ice-cold PBS. Biotin was removed from proteins remaining at the cell surface by incubation with a solution containing 20 mM sodium 2-mercaptoethanesulfonate (MesNa) in 50 mM Tris-HCl (pH 8.6), 100 mM NaCl for 1 h at 4 °C. MesNa was quenched by the addition of 20 mM iodoacetamide (IAA) for 10 min, and after other two further washes in PBS, the cells were lysed in 25 mM Tris-HCl, pH 7.4, 100 mM NaCl, 2 mM MgCl_2_, 1 mM Na_3_VO_4_, 0.5 mM EGTA, 1% Triton X-100, 5% glycerol, protease mix (Sigma), and 1 mM PMSF. Lysates were cleared by centrifugation at 12,000*g* for 20 min. Supernatants were corrected to equivalent protein concentrations by BCA assay, and integrins were isolated by immunoprecipitation and analyzed by SDS-PAGE.

### RNA isolation and reverse transcription.

Cells were placed in RNA*later* solution (Ambion), kept at 4 °C for 24 h, and frozen at −80 °C. After the cells were thawed on ice, total RNA was extracted following the manufacturer's recommended protocol (SV Total RNA Isolation System, Promega). The quality and integrity of the total RNA were quantified by means of the RNA 6000 Nano Assay kit in an Agilent 2100 bioanalyzer (Agilent Technologies). cDNAs were generated from 1 μg of total RNA using the High Capacity cDNA Reverse Transcription Kit (Applied Biosystems).

### TaqMan real-time RT-PCR assay.

mRNA expression of FN and endogenous control genes, i.e., 18S rRNA, glyceraldehyde 3-phosphate dehydrogenase (GAPDH), and TATA binding protein (TBP), was measured in the samples by real-time RT-PCR using TaqMan Gene Expression Assays run on an ABI PRISM 7900HT Fast Real-Time PCR System (Applied Biosystems). The following assays were used: Hs00365058_m1 (FN), Hs99999901_s1 (18S rRNA), Hs99999905_m1 (GAPDH), and Hs00427620_m1 (TBP). Three replicates were run for each gene for each sample in a 384-well format plate (cDNA concentration 20 ng/well) according the manufacturer's protocol. Between the three measured endogenous control genes, we chose TBP for normalization, identified by geNorm [74]. The experimental threshold (Ct) was calculated using the algorithm provided by the SDS 1.9.1 software (Applied Biosystems). Ct values were converted into relative quantities using the method described here [75]. The amplification efficiency of each gene was calculated using a dilution curve and the slope calculation method [75].

### Conventional confocal scanning microscopy.

Cells were plated on glass coverslips coated with 1 μg/ml FN (TebuBio) and allowed to adhere for 3 h. In addition, ECs cotransfected with *m*Nrp1-mRFP and Rab5-GFP were then washed in PBS, incubated with 10 μg/ml SNAKA51 Ab in EBM-2 for 30 min at 4 °C, washed 3 times in PBS, transferred to prewarmed EGM-2, and allowed to recover at 37 °C for 2 min to induce endocytosis. Cells were washed in PBS, fixed in 4% paraformaldehyde, permeabilized in 0.01% saponin for 10 min on ice, and incubated or not with the Alexa-Fluor-405-conjugated secondary antibody (Molecular Probes) for 1 h at room temperature. Cells were analyzed by using a Leica TCS SP2 AOBS confocal laser-scanning microscope (Leica Microsystems). Immunofluorescence analysis was performed as previously described [16].

### Fibronectin fibrillogenesis.

Small interfering RNA silencing was performed, and after the second oligofection, cells were seeded onto glass coverslips in six-well dishes at a concentration of 20 × 10^4^ cells/well and left to adhere for 3 h in EGM-2 medium (Clonetics) containing FN-depleted serum. Cells were then washed with PBS and fixed with 3.7% paraformaldehyde for 20 min at room temperature. Next, cells were permeabilized in PBS containing 0.1% Triton X-100 on wet ice for 2 min and incubated with anti-FN Ab for 1 h at room temperature. After three washes, cells were incubated with anti-mouse Alexa Fluor 555 for 45 min at room temperature and subsequently with DAPI. Cells were finally examined using a Leica TCS SP2 AOBS confocal laser-scanning microscope (Leica Microsystems).

### FRET detection by TIRF/FLIM analysis.

Fluorescence resonance energy transfer was detected using a Lambert Instruments fluorescence attachment (LIFA) on a Nikon Eclipse TE 2000-U microscope, with the same changes to the condenser as described above and a filter block consisting of a Z473/10 excitation filter, a Z 488 RDC dichroic mirror, and a HQ 525/50M emission filter. The light source was a modulated 473-nm laser diode, which allows, in combination with the modulated intensifier from the LIFA system, measurement of fluorescence lifetimes using frequency domain. The laser was brought into TIRF mode before acquiring the images for the lifetime analysis.

Donor (D) lifetime, τ, was analyzed either in the presence or in the absence of the acceptor (A), in adhesion sites, characterized by high donor concentrations, using the FLIM software (version 1.2.1.1.130; Lambert Instruments, The Netherlands). Fluorescence resonance energy transfer efficiency (E) was calculated as E = 1 – (τDA/τD). Lifetime τ was evaluated in four different areas (12 × 12 pixels) of seven α5-GFP and seven α5-GFP/*m*Nrp1-Cherry transfected NIH 3T3 cells. For statistical evaluation, results were analyzed with Student's *t* test.

### TIRF-based photoactivation and time-lapse microscopy.

Total internal reflection fluorescence experiments have been performed on a Nikon Eclipse TE 2000-U microscope equipped with 60× and 100× 1.45 NA Nikon TIRF oil immersion objectives. The Nikon Epi-fluorescence condenser was replaced with a custom condenser in which laser light was introduced into the illumination pathway directly from the optical fiber output oriented parallel to the optical axis of the microscope. The light source for evanescent wave illumination was either a 473-nm diode, a 405-nm diode, or a 561-nm laser (Omicron), with each laser line coupled into the condenser separately to allow individual TIRF angle adjustments. Each laser was controlled separately by a DAC 2000 card or a uniblitz shutter operated by MetaMorph (Molecular Devices). A filter block consisting of an E480SPX excitation filter, a FF 495 dichroic mirror, and an ET 525/50M emission filter was used for activation of α5-PA-GFP with the 405-nm laser. After activation the filter was manually changed to a green/red dual filter block (ET-GFP/mCherry from AHF Analysentechnik, Germany) to allow simultaneous time-lapse acquisition of activated α5-PA-GFP and *m*Nrp1-Cherry using 473- and 561-nm excitation. A Multi-Spec dual emission splitter (Optical Insights, NM) with a 595-nm dichroic and two bandpass filters (510–565 nm for green and 605–655 nm for red) was used to separate both emissions. All cell imaging was performed with a Cascade 512F EMCCD camera (Photometrics UK).

### Confocal photoactivation.

Localized activation of α5-PA-GFP in *m*Nrp1-Cherry-positive vesicles was done on a FV 1000 Olympus confocal microscope, using two-channel imaging and a separate SIM scanner for 405-nm activation [51].

## Supporting Information

Figure S1 Analysis of the Influence of *h*Nrp1, *h*GIPC1, and *h*Myo6 Silencing on FN mRNA, protein levels, and Fibrillogenesis (A–F) Real-time RT-PCR (A–C) and Western blot analyses (D–F) on total RNAs and proteins extracted at different times of cell spreading in the absence of exogenously added extracellular matrices reveal that *h*Myo6 (C,F), but neither *h*Nrp1 (A,D) nor *h*GIPC1 (B,E), silencing reduce FN mRNA and protein levels. Vinculin was used as normalizer protein in Western blot analysis to calculate normalized optical density (NOD) units.
(G–I) Confocal scanning microscopy analysis of endogenous FN fibrils in siCtl (G), si*h*GIPC1 (H), or si*h*Myo6 (I) transfected ECs. DAPI was used to stain nuclei. White bar in (I) corresponds to 50 μm.
(10.0 MB TIF)

Figure S2 Neither Full-Length *m*Nrp1 nor Its Deletion Constructs Modulates Cell Adhesion to VN *m*Nrp1, *m*Nrp1dSEA, or *m*Nrp1dCy overexpression does not affect the adhesion of NIH 3T3 fibroblasts to VN.
(825 KB TIF)

Figure S3 Nrp1 Silencing Does Not Affect Either Total or Active α5β1 Integrin Levels in ECs Immunoprecipitation of either total or active α5β1 followed by Western blot analysis for α5 shows that silencing Nrp1 in human ECs (si*h*Nrp1) does not alter α5 integrin expression compared with that of control silenced cells (siCtl).
(1.5 MB TIF)

Figure S4 Rapid α5β1 Integrin Turnover in ECM Adhesions As in Figure 7, α5-PA-GFP was photoactivated in TIRF in NIH 3T3 cells and observed in time-lapse epifluorescence microscopy. Immediately after photoactivation, the α5-PA-GFP signal starts leaving the adhesive sites and accumulating in vesicles and disappears by ∼45 s in about 50% of the adhesion sites and by ∼115 s in the remaining ones (see also Video S2).
(9.6 MB TIF)

Figure S5 Upon Photoactivation in Areas outside of *m*Nrp1-Cherry-Positive Vesicles, α5-PA-GFP Does Not Recycle Back to Membrane Adhesions NIH 3T3 cells were cotransfected with *m*Nrp1-Cherry and α5-PA-GFP. α5-PA-GFP integrin fluorescence was then locally photoactivated in an *m*Nrp1-Cherry-positive area devoid of vesicles (blue circle) and followed in time-lapse confocal microscopy. Fluorescence intensity was measured over time outside of Nrp1-positive vesicles (blue circle) and at the plasma membrane (red circle). The time-lapse plot (lower panel) shows that, under the same experimental conditions used in the experiment shown in Figure 8, little or no photoactivation of α5-PA-GFP occurred, and no fluorescence intensity increase was detected at the plasma membrane (see also Video S4).
(9.4 MB TIF)

Video S1 Rapid Turnover of α5β1 Integrin from Adhesion Sites into Nrp1-Positive Vesicles As in Figure 7, a representative NIH 3T3 cell was cotransfected with *m*Nrp1-Cherry and α5-PA-GFP, photoactivated in TIRF, and observed in time-lapse epifluorescence microscopy.
(4.3 MB MOV)

Video S2 Rapid α5β1 Integrin Turnover in ECM Adhesions: Time-Lapse Epifluorescence Microscopy of α5-PA-GFP in the Representative NIH 3T3 Cell Shown in Figure S4
(3.1 MB MOV)

Video S3 Upon Photoactivation in *m*Nrp1-Cherry-Positive Vesicles, α5-PA-GFP Recycles Back to Membrane Adhesions As in Figure 8, a representative NIH 3T3 cell was cotransfected with *m*Nrp1-Cherry and α5-PA-GFP. α5-PA-GFP was locally photoactivated in vesicles containing *m*Nrp1-Cherry and followed by time-lapse confocal microscopy.
(9.7 MB MOV)

Video S4 Upon Photoactivation in Areas outside of *m*Nrp1-Cherry-Positive Vesicles, α5-PA-GFP Does Not Recycle Back to Membrane Adhesions As in Figure S5, a representative NIH 3T3 cell was cotransfected with *m*Nrp1-Cherry and α5-PA-GFP. α5-PA-GFP was locally photoactivated in an *m*Nrp1-Cherry-positive area devoid of vesicles and followed by time-lapse confocal microscopy.
(2.6 MB MOV)

## Abbreviations

α5-GFP: GFP-tagged α5 integrin subunit
α5-PA-GFP: photoactivatable α5-GFP
τ: lifetime
A: acceptor
Ab: antibody
Cherry: an improved version of mRFP
CHO B2: CHO cells lacking the α5 integrin subunit
CHO B2α27: CHO cells expressing the α5 integrin subunit
COLL-I: type I collagen
CUB: complement-binding
D: donor
E: FRET efficiency
ECs: endothelial cells
ECM: extracellular matrix
FLIM: fluorescence lifetime imaging microscopy
FN: fibronectin
*FN1,*: human fibronectin 1 gene
FRET: fluorescence resonance energy transfer
GFP: green fluorescent protein
GIPC1: GAIP interacting protein C terminus, member 1
HA: hemagglutinin
*h*Nrp1: human full-length Nrp1
LN: laminin
mAb: monoclonal Ab
MAM: meprin/A5/μ-phosphatase
*m*Nrp1: full-length mouse Nrp1
*m*Nrp1-Cherry: Cherry-tagged mNrp1
*m*Nrp1dCy: murine Nrp1 lacking the whole cytoplasmic domain
*mNrp1dSEA*: murine Nrp1 lacking the C-terminal SEA amino acids
*m*Nrp1-mRFP: mRFP-tagged mNrp1
mRFP: monomeric red fluorescent protein
Myo6: myosin 6
NOD: normalized optical density units
PMQ: primaquine
RNAi: RNA interference
Sema: murine semaphorin
SEMA: human semaphorin
SEMA3: class 3 SEMA
sFN: soluble FN dimers
siCtl: control nontargeting siRNA
si*h*GIPC1: siRNAs targeting human GIPC1
si*h*Myo6: siRNAs targeting human Myo6
si*h*Nrp1: siRNAs targeting *h*Nrp1
TIRF: total internal reflection fluorescence
VEGF: vascular endothelial growth factor
VN: vitronectin
