# Expression of Concern: Neuropilin-1/GIPC1 Signaling Regulates α5β1 Integrin Traffic and Function in Endothelial Cells

**DOI:** 10.1371/journal.pbio.3001840

**Published:** 2022-10-05

**Authors:** The PLOS Biology Editors

After publication of this article [1], concerns were raised about potential lane similarities and discontinuities in the top left western blot panel in Fig 10F (IP: total α5β1, results for Membrane expression and the 0 timepoint). Specifically:

- Lane 1 appears similar to lane 2.
- When levels are adjusted to visualize background details, there appear to be vertical discontinuities on either side of lane 2.

**Fig 10:**
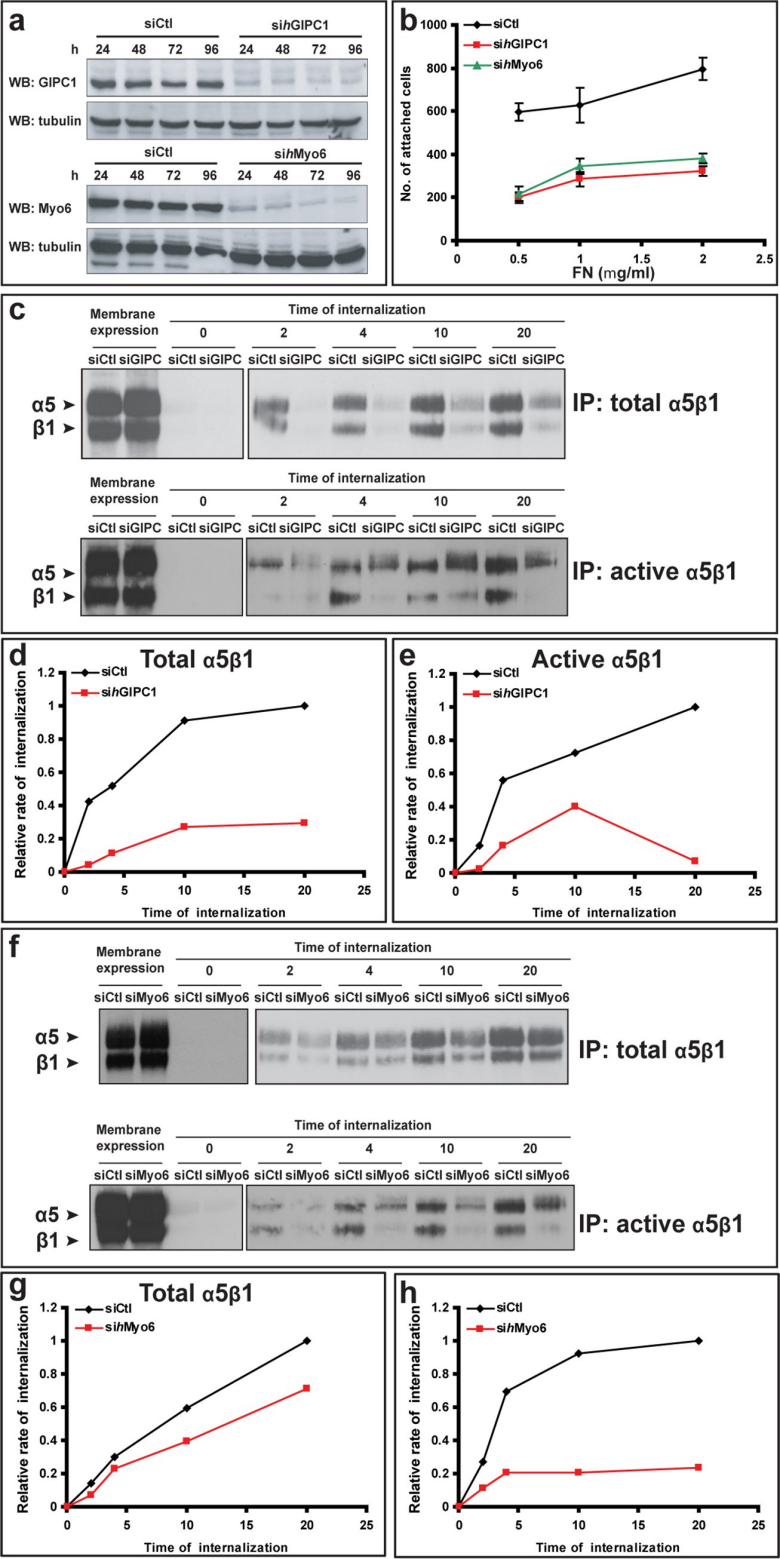
In ECs GIPC1 and Myo6 Regulate α5β1 Integrin Traffic and Function (A) Western blot analysis of protein expression in ECs silenced for human GIPC1 (si*h*GIPC1) or Myo6 (si*h*Myo6) or transfected with control siRNA (siCtl) reveals an efficient silencing of GIPC1 or Myo6 at 96 h after the second oligofection. (B) Comparison between siCtl (black) and either si*h*GIPC1 (red) or si*h*Myo6 (green) transfected ECs adhering to FN. (C) Time-course analysis reveals an impairment of both total and active α5β1 integrin internalization in ECs silenced for *h*GIPC1 in comparison with control cells (siCtl). (D,E) Relative quantification of time-lapse endocytosis assay (shown in (C)) of total (D) or active (E) α5β1 integrin in ECs silenced for *h*GIPC1. (F) Time-course analysis reveals a significant impairment of active but not total α5β1 integrin internalization in ECs silenced for *h*Myo6 in comparison with control cells (siCtl). (G,H) Relative quantification of time-course endocytosis assay (shown in (F)) of total (G) or active (H) α5β1 integrin in ECs silenced for *h*Myo6.

The first and corresponding authors stated that an error was made in assembling this figure panel and the wrong blot was included. They further stated that they have not been able to recover the original data for the top left western blot panel in question.

The authors provided a corrected version of Fig 10 in which the top left western blot panel was replaced (see below), which they state, includes the correct data from the original experiment. However, the authors have been unable to provide the original data underlying the top left western blot panel that was originally published in Fig 10F, although they have provided all data underlying the remainder of the original Fig 10 panels, as well as those underlying the corrected top left western blot panel in Fig 10F (S1–S3 Files).

The first and corresponding authors stated that the correction of the upper left western blot of panel F of Fig 10 does not change the finding, documented in the original upper right western blot, that silencing of Myosin 6 only modestly influences the endocytosis of total α5β1 integrin in endothelial cells.

*PLOS Biology* concluded that the results reported for the Fig 10F experiment are adequately supported by the data reported for timepoints 2–20 (upper right blot). However, in light of the unavailability of the underlying data for the original top left western blot in panel F of Fig 10, the concerns about this western blot remain unresolved and so the *PLOS Biology* Editors issue this Expression of Concern.

## Supporting information

S1 File Scan of the control surface biotinylation western blot underlying the top left western blot panel in the corrected Fig 10F. (TIF)

S2 File Scans of underlying western blots supporting the original Fig 10A and 10C and 10F excluding the original top left western blot panel in Fig 10F. (ZIP)

S3 File Underlying data supporting the charts in the original Fig 10B, 10D, 1E, 10G and 10H. (XLSX)
