# Supplementary figures and images for: Neuropilin-1/GIPC1 Signaling Regulates α5β1 Integrin Traffic and Function in Endothelial Cells
Source: PLoS Biol. 2009 Jan 27;7(1):e1000025. doi: 10.1371/journal.pbio.1000025 (PMC2631072; doi:10.1371/journal.pbio.1000025)

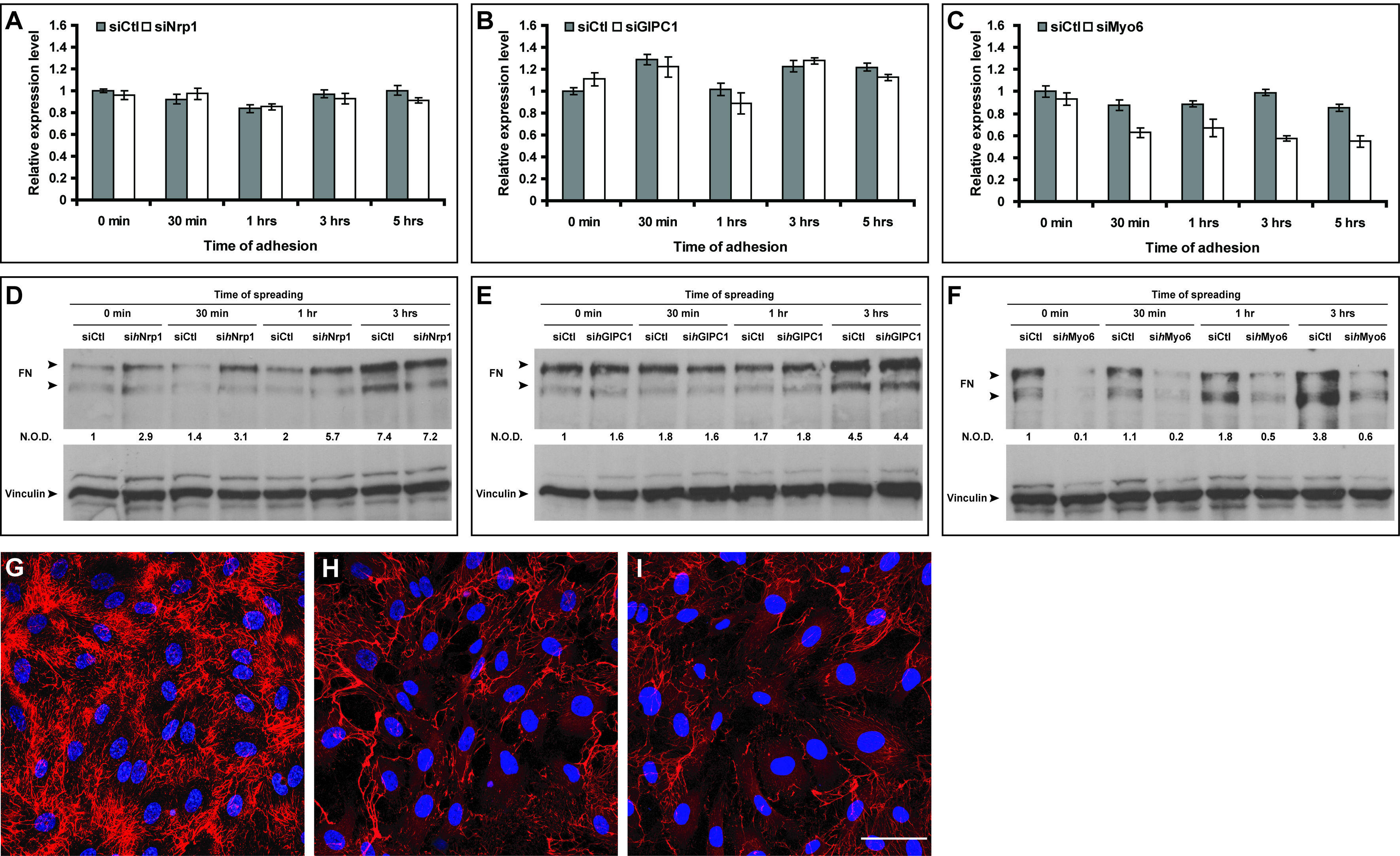

Supplement: Figure S1 — (A–F) Real-time RT-PCR (A–C) and Western blot analyses (D–F) on total RNAs and proteins extracted at different times of cell spreading in the absence of exogenously added extracellular matrices reveal that hMyo6 (C,F), but neither hNrp1 (A,D) nor hGIPC1 (B,E), silencing reduce FN mRNA and protein levels. Vinculin was used as normalizer protein in Western blot analysis to calculate normalized optical density (NOD) units. (G–I) Confocal scanning microscopy analysis of endogenous FN fibrils in siCtl (G), sihGIPC1 (H), or sihMyo6 (I) transfected ECs. DAPI was used to stain nuclei. White bar in (I) corresponds to 50 μm. (10.0 MB TIF) [file pbio.1000025.sg001.tif]

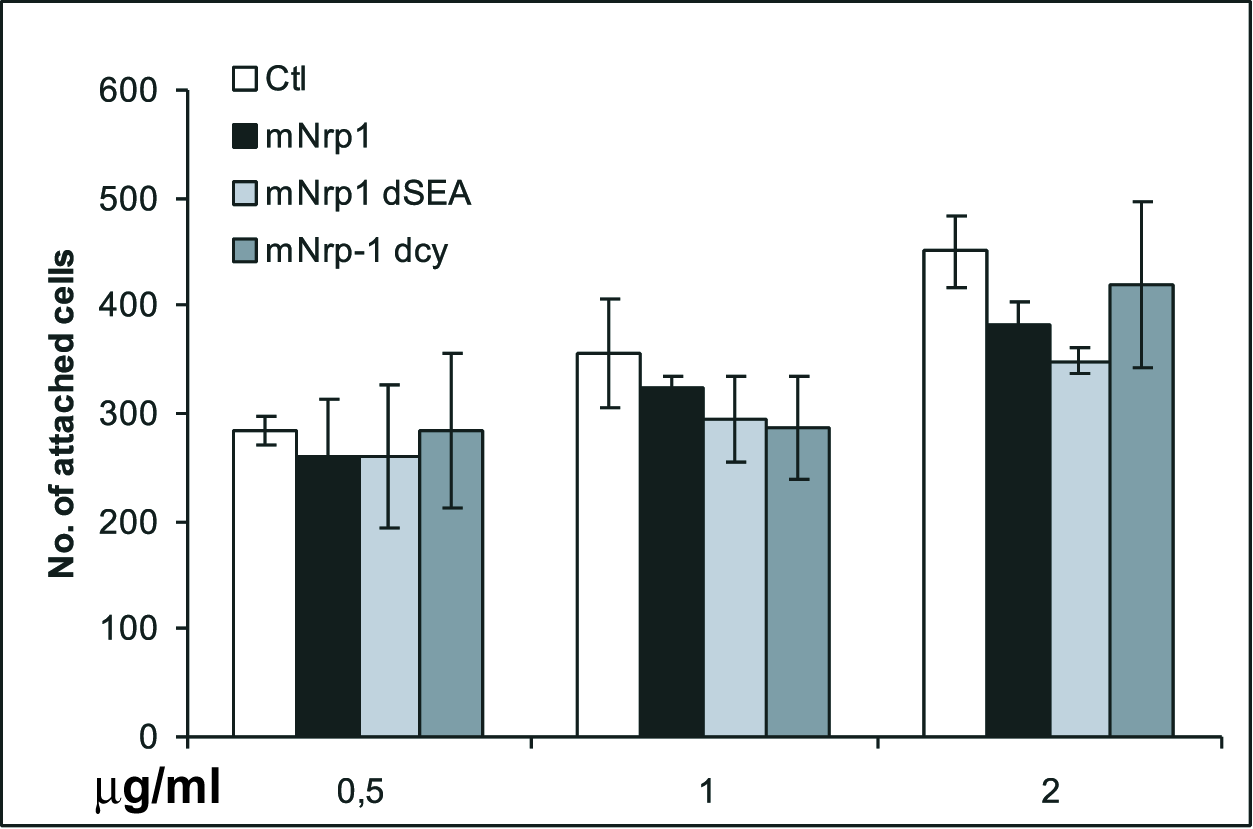

Supplement: Figure S2 — mNrp1, mNrp1dSEA, or mNrp1dCy overexpression does not affect the adhesion of NIH 3T3 fibroblasts to VN. (825 KB TIF) [file pbio.1000025.sg002.tif]

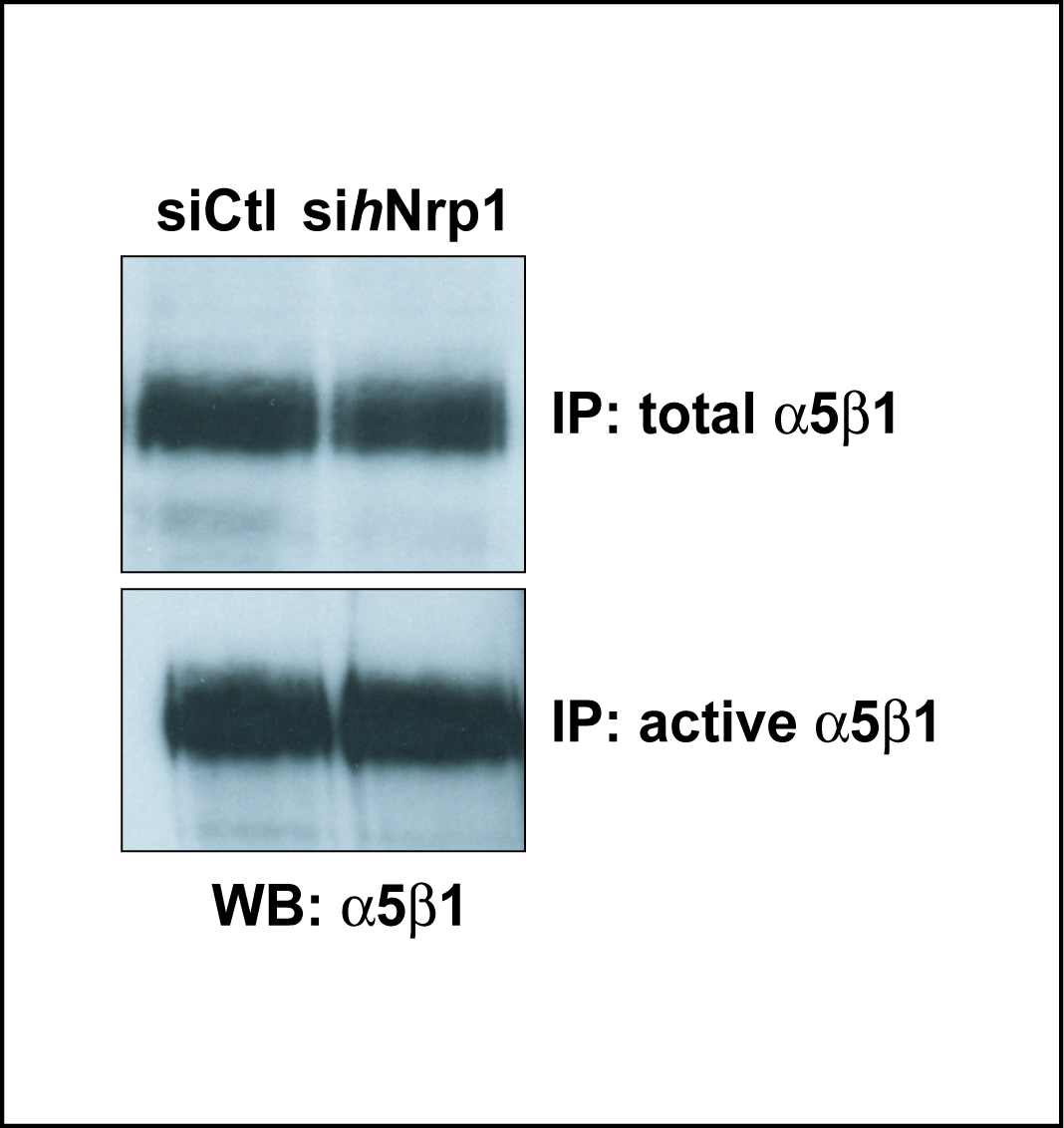

Supplement: Figure S3 — Immunoprecipitation of either total or active α5β1 followed by Western blot analysis for α5 shows that silencing Nrp1 in human ECs (sihNrp1) does not alter α5 integrin expression compared with that of control silenced cells (siCtl). (1.5 MB TIF) [file pbio.1000025.sg003.tif]

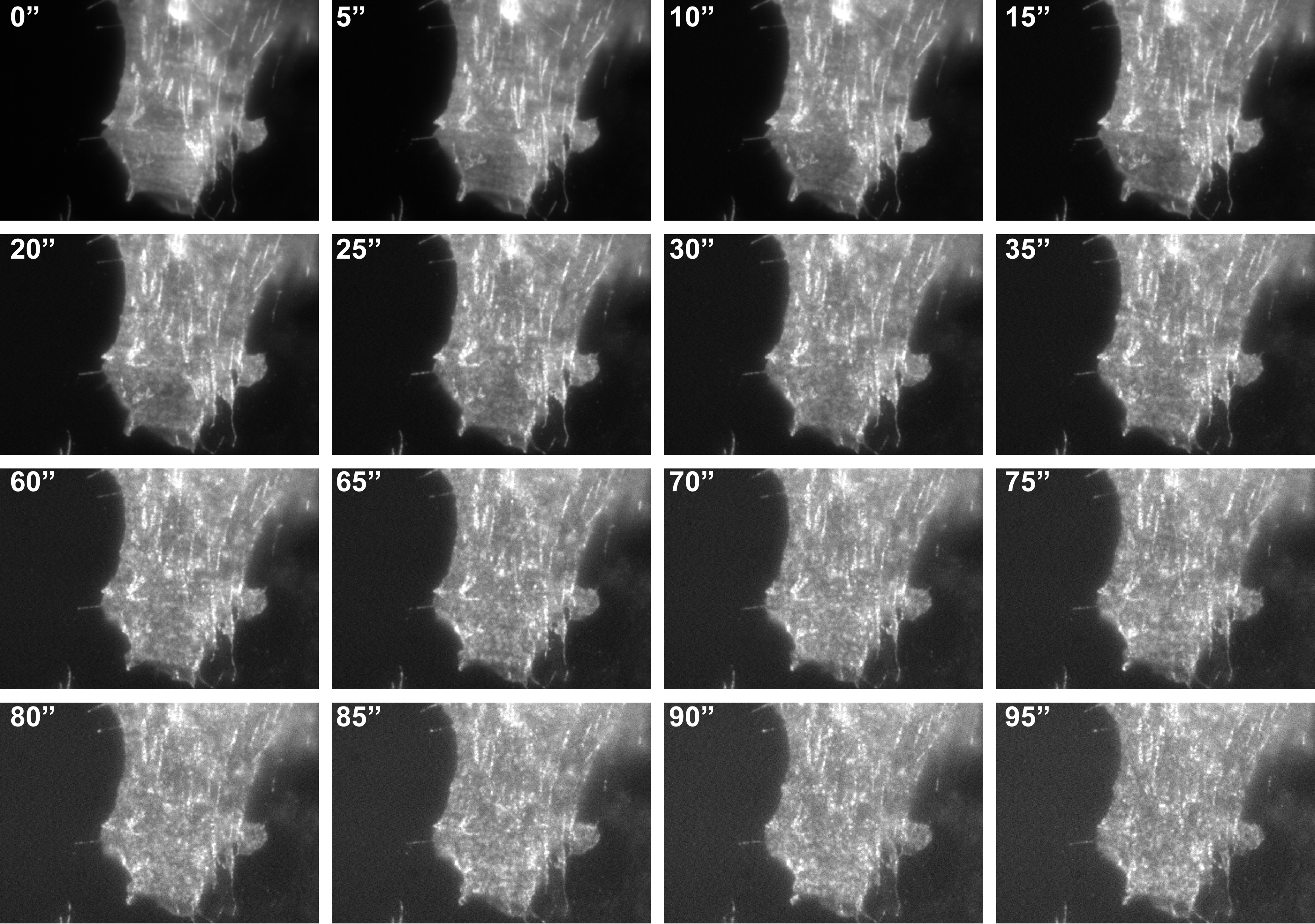

Supplement: Figure S4 — As in Figure 7, α5-PA-GFP was photoactivated in TIRF in NIH 3T3 cells and observed in time-lapse epifluorescence microscopy. Immediately after photoactivation, the α5-PA-GFP signal starts leaving the adhesive sites and accumulating in vesicles and disappears by ∼45 s in about 50% of the adhesion sites and by ∼115 s in the remaining ones (see also Video S2). (9.6 MB TIF) [file pbio.1000025.sg004.tif]

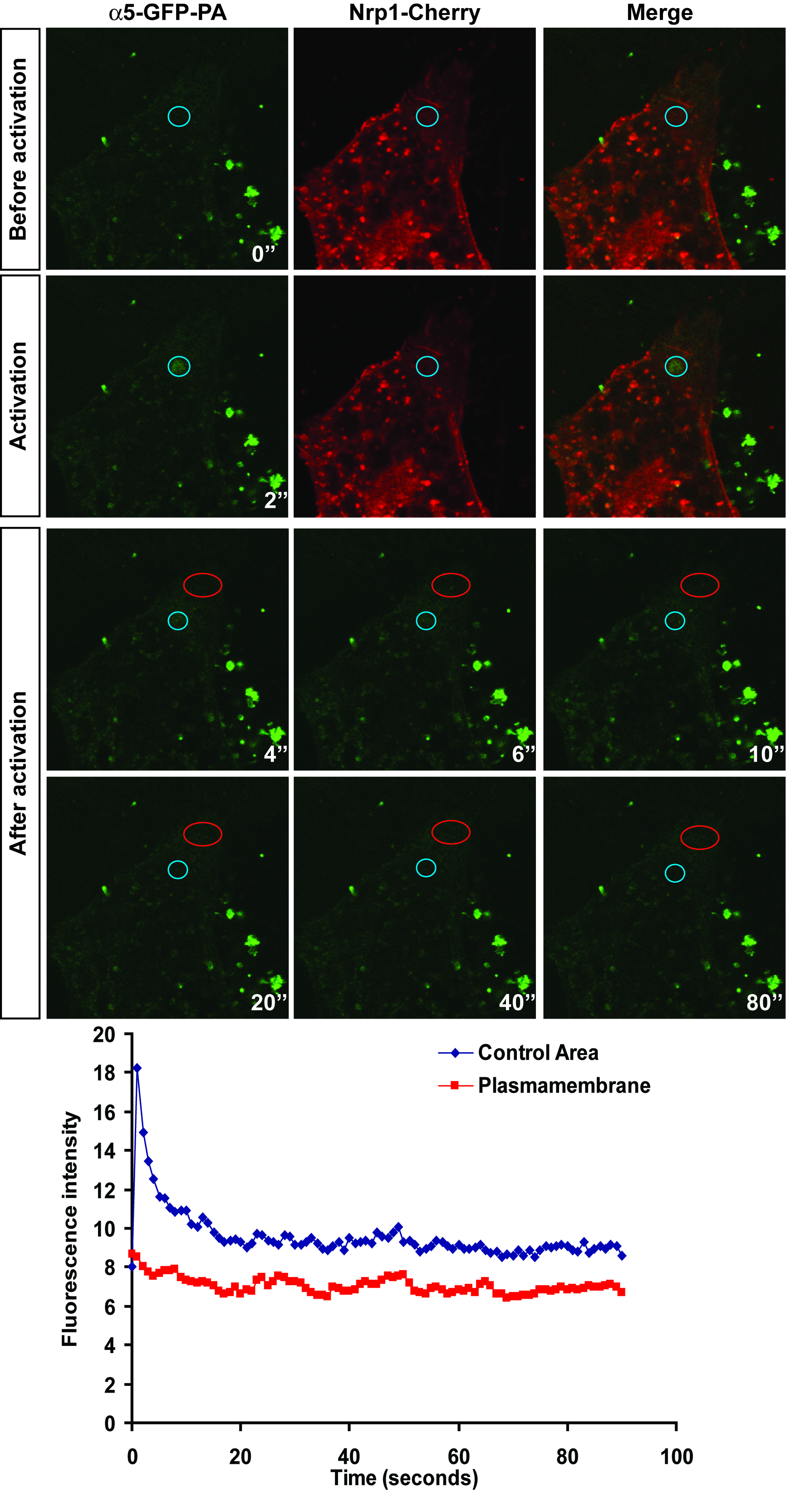

Supplement: Figure S5 — NIH 3T3 cells were cotransfected with mNrp1-Cherry and α5-PA-GFP. α5-PA-GFP integrin fluorescence was then locally photoactivated in an mNrp1-Cherry-positive area devoid of vesicles (blue circle) and followed in time-lapse confocal microscopy. Fluorescence intensity was measured over time outside of Nrp1-positive vesicles (blue circle) and at the plasma membrane (red circle). The time-lapse plot (lower panel) shows that, under the same experimental conditions used in the experiment shown in Figure 8, little or no photoactivation of α5-PA-GFP occurred, and no fluorescence intensity increase was detected at the plasma membrane (see also Video S4). (9.4 MB TIF) [file pbio.1000025.sg005.tif]
