# Supplementary figures and images for: Expression of Concern: Neuropilin-1/GIPC1 Signaling Regulates α5β1 Integrin Traffic and Function in Endothelial Cells
Source: PLoS Biol. 2022 Oct 5;20(10):e3001840. doi: 10.1371/journal.pbio.3001840 (PMC9534557; doi:10.1371/journal.pbio.3001840)

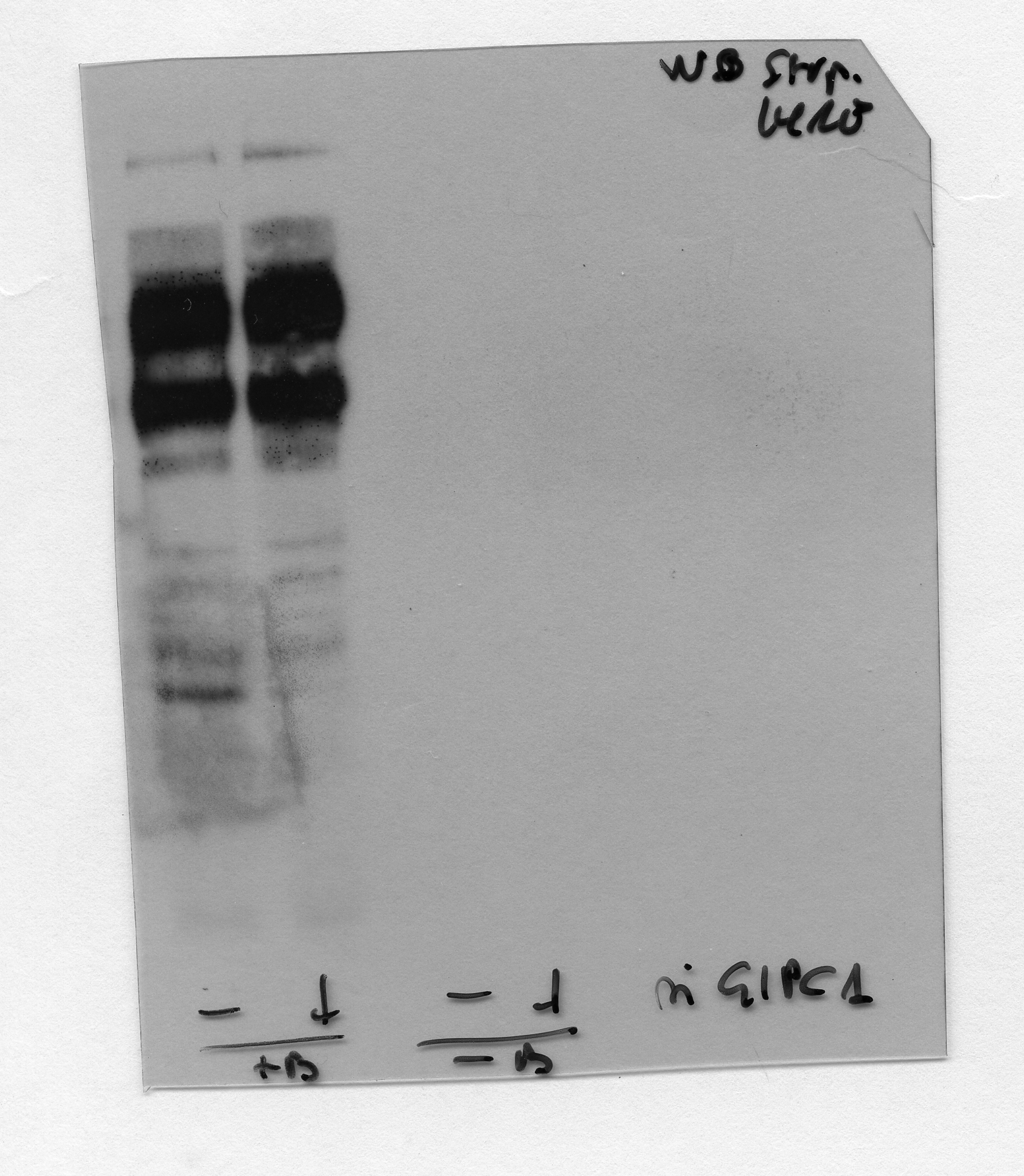

Supplement: S2 File — (ZIP) [file pbio.3001840.s002.zip › Valdembri et al_Original WBs Figure 10_Panel C bottom left.tif]

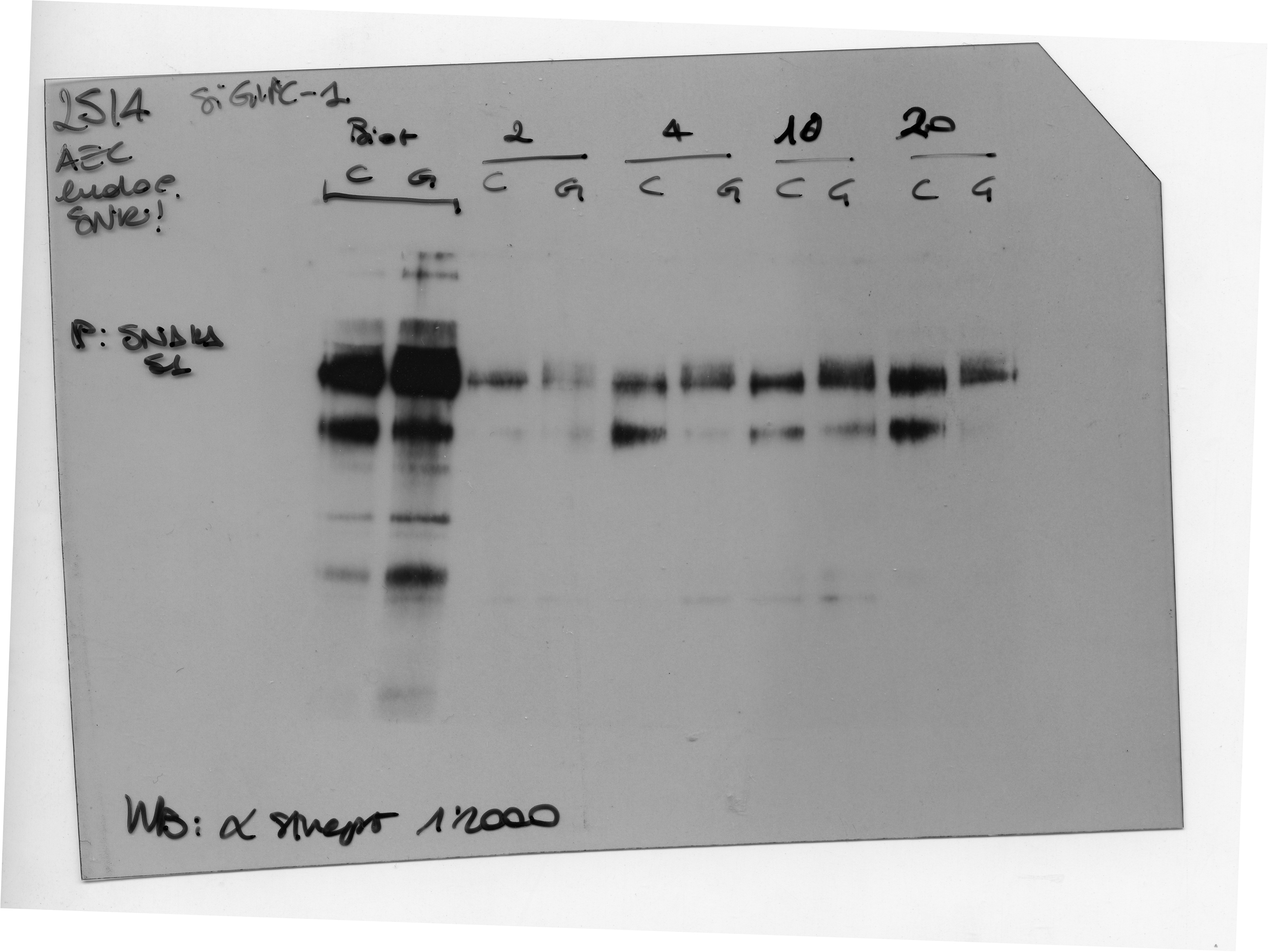

Supplement: S2 File — (ZIP) [file pbio.3001840.s002.zip › Valdembri et al_Original WBs Figure 10_Panel C bottom right.tif]

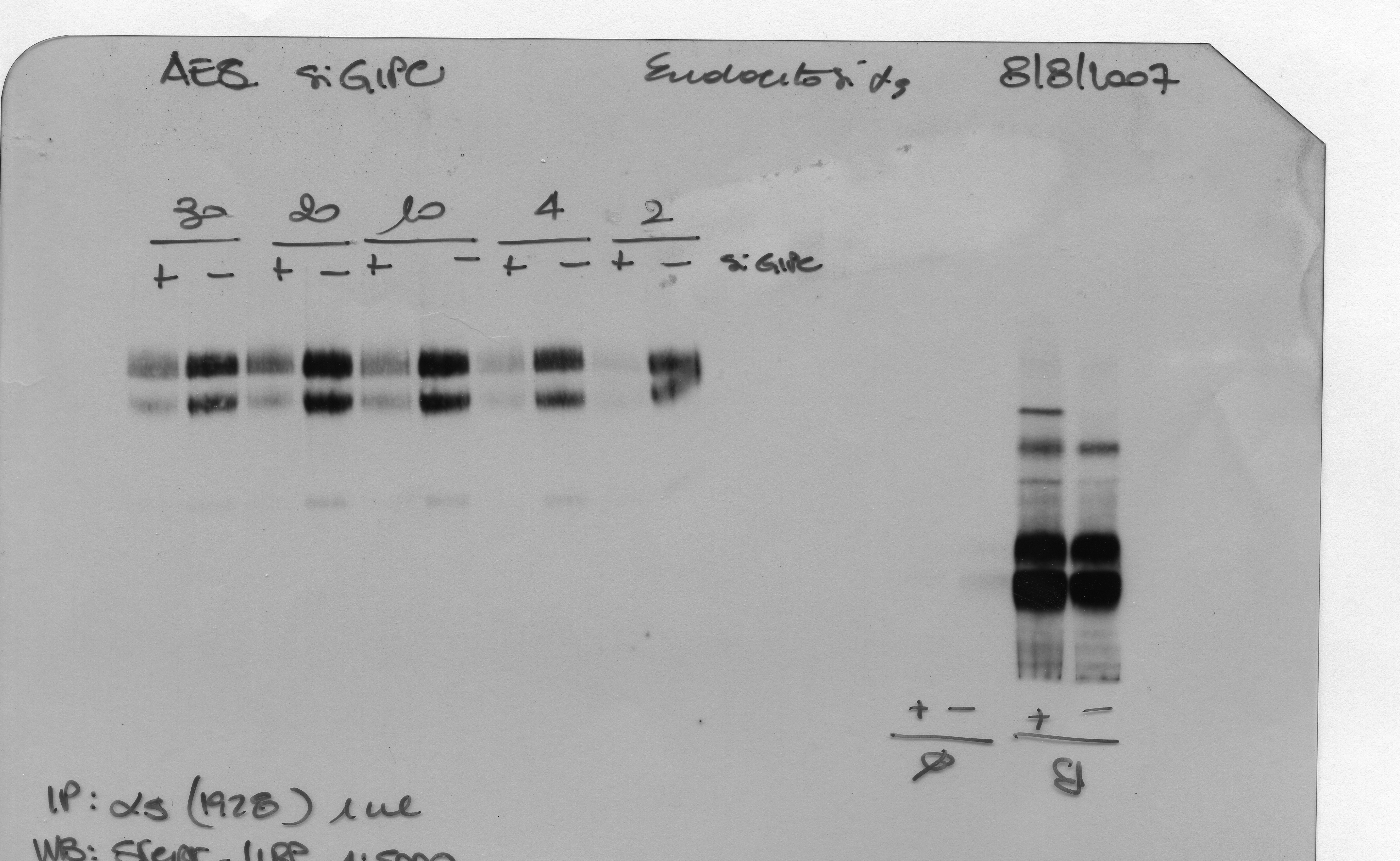

Supplement: S2 File — (ZIP) [file pbio.3001840.s002.zip › Valdembri et al_Original WBs Figure 10_Panel C up two panels.tif]

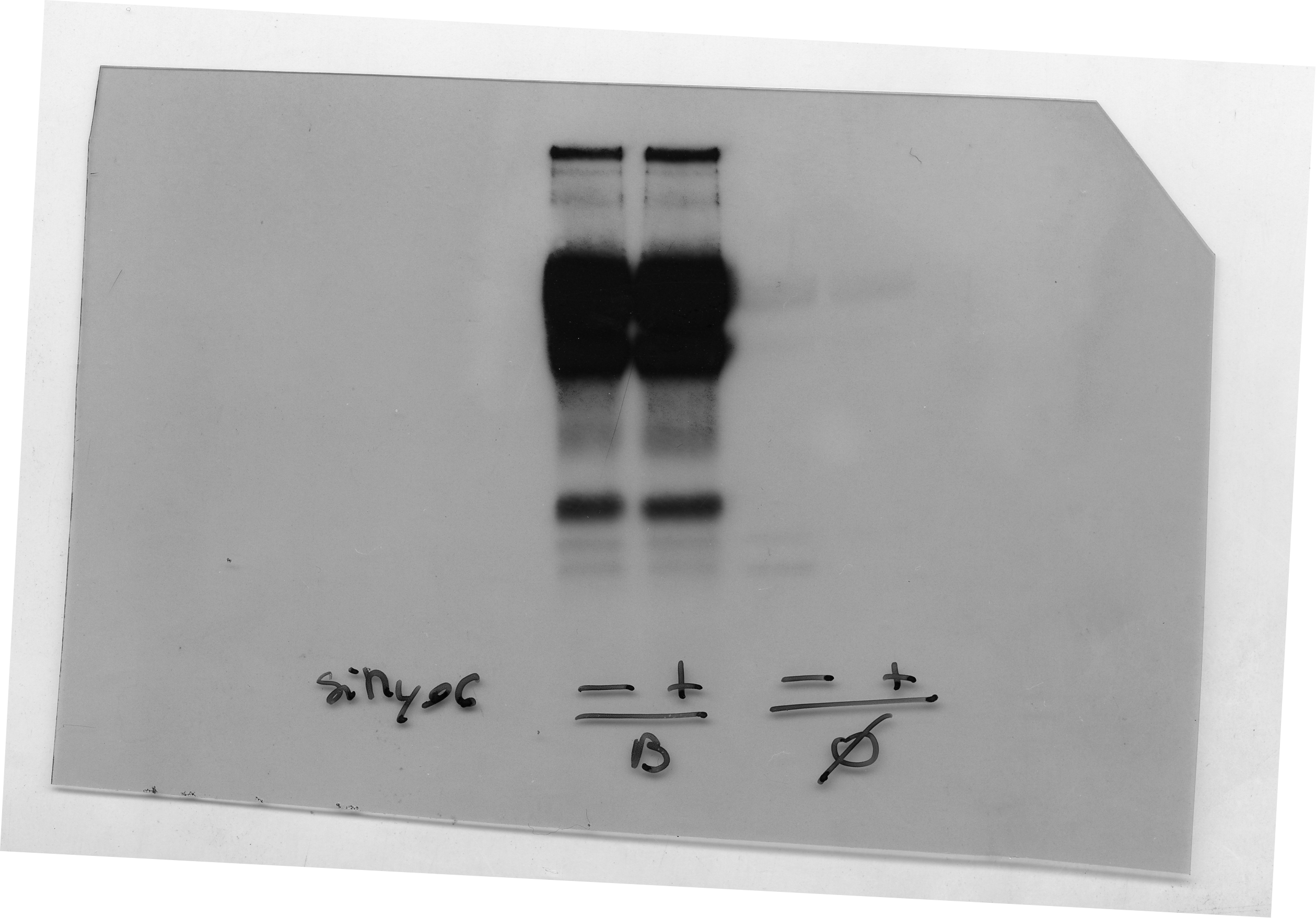

Supplement: S2 File — (ZIP) [file pbio.3001840.s002.zip › Valdembri et al_Original WBs Figure 10_Panel F bottom left.tif]

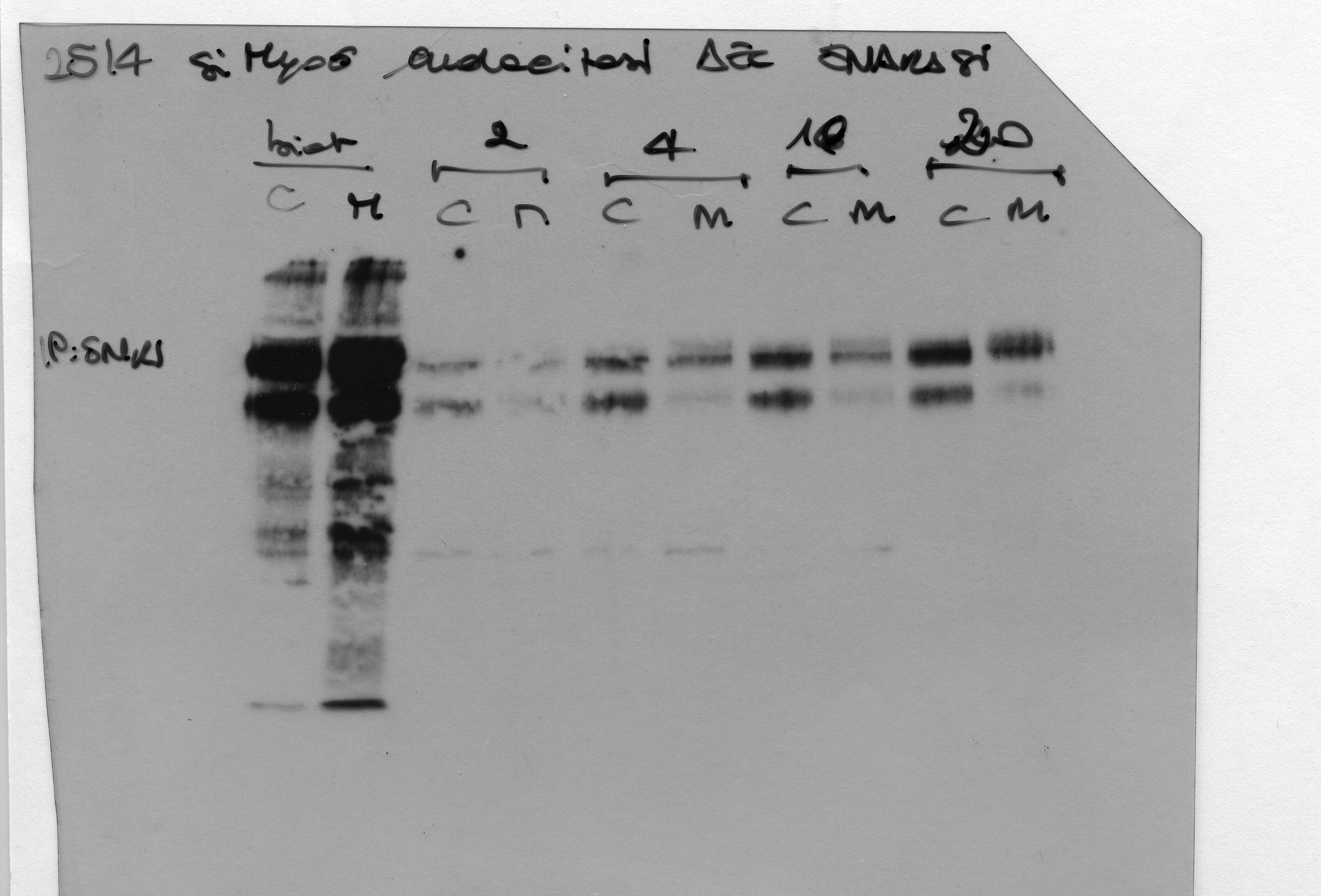

Supplement: S2 File — (ZIP) [file pbio.3001840.s002.zip › Valdembri et al_Original WBs Figure 10_Panel F bottom right.tif]

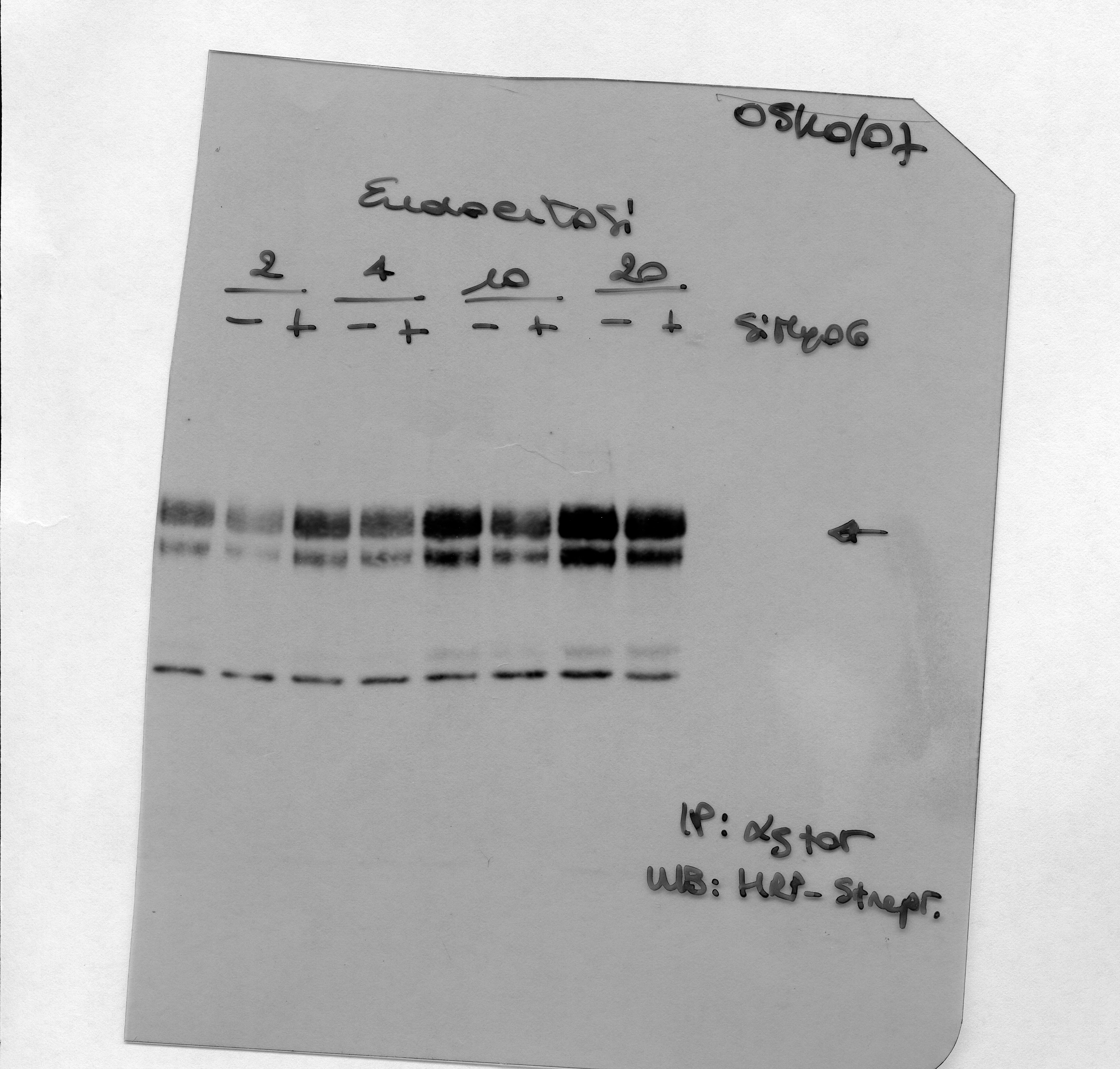

Supplement: S2 File — (ZIP) [file pbio.3001840.s002.zip › Valdembri et al_Original WBs Figure 10_Panel F top right.tif]
